# Integrating metagenomics and metabolomics to study the gut microbiome and host relationships in sports across different energy systems

**DOI:** 10.1038/s41598-025-98973-2

**Published:** 2025-05-02

**Authors:** Viviana Aya, Daniel Pardo-Rodriguez, Laura Camila Vega, Mónica P. Cala, Juan David Ramírez

**Affiliations:** 1 Centro de Investigaciones en Microbiología y Biotecnología-UR (CIMBIUR), Facultad de Ciencias Naturales, Universidad del Rosario, Bogotá, Colombia; 2 MetCore - Metabolomics Core Facility, Vice-Presidency for Research, Universidad de los Andes, Bogotá, Colombia; 3 Molecular Microbiology Laboratory, Department of Pathology, Molecular and Cell-Based Medicine, Icahn School of Medicine at Mount Sinai, New York, NY USA

**Keywords:** Gut microbiome, Athletes, Metabolomics, Metagenomics, Lipidomics, Sports, Metagenomics, Biochemistry, Microbiology

## Abstract

The gut microbiome plays a critical role in modulating host metabolism, influencing energy production, nutrient utilization, and overall physiological adaptation. In athletes, these microbial functions may be further specialized to meet the unique metabolic demands of different sports disciplines. This study explored the role of the gut microbiome in modulating host metabolism among Colombian athletes by comparing elite weightlifters (n = 16) and cyclists (n = 13) through integrative omics analysis. Fecal and plasma samples collected one month before an international event underwent metagenomic, metabolomic, and lipidomic profiling. Metagenomic analysis revealed significant microbial pathways, including L-arginine biosynthesis III and fatty acid biosynthesis initiation. Key metabolic pathways, such as phenylalanine, tyrosine, and tryptophan biosynthesis; arginine biosynthesis; and folate biosynthesis, were enriched in both athlete groups. Plasma metabolomics and lipidomics revealed distinct metabolic profiles and a separation between athlete types through multivariate models, with lipid-related pathways such as lipid droplet formation and glycolipid synthesis driving the differences. Notably, elevated carnitine, amino acid, and glycerolipid levels in weightlifters suggest energy system-specific metabolic adaptations. These findings underscore the complex relationship between the gut microbiota composition and metabolic responses tailored to athletic demands, laying the groundwork for personalized strategies to optimize performance. This research highlights the potential for targeted modulation of the gut microbiota as a basis for tailored interventions to support specific energy demands in athletic disciplines.

## Introduction

The gut microbiota of physically active individuals has been thoroughly described, revealing a greater prevalence of beneficial microorganisms^1–3^; specifically, the gut microbiota of athletes has been characterized by a higher prevalence of butyrate-producing bacteria, such as *Eubacterium rectale*^4^, *Akkermansia muciniphila*^5^*, Eubacterium hallii*^6,7^, and *Faecalibacterium prausnitzi*^8,9^, among others^10^. Metagenomic data from highly trained subjects revealed the presence of numerous bacterial families involved in crucial metabolic functions^11^. Recent research has shown that exercise leads to elevated levels of lysophosphatidylcholine species synthesized by the gut microbiota, leading to increased lipid oxidation and reduced activity in metabolic pathways associated with oxidative stress^12^.

Most of the research in this field has focused on endurance training, particularly aerobic exercise^13,14^, which is key for energy production via the oxidative system^1,15^. Endurance athletes, such as cyclists and runners, demonstrate exceptional cardiovascular fitness and enhanced oxidative metabolism. While many studies have examined the role of the microbiome in these activities^16–18^, the effects of strength and other exercise forms on the gut microbiota remain underexplored. The inclusion of athletes from various backgrounds could reveal the diverse functional capabilities of the gut microbiome and its reciprocal relationship with exercise. Traditionally, exercise metabolism has been divided into glycolytic and oxidative pathways, reflecting the energy systems engaged during different exercise intensities and durations. However, recent research has suggested that the metabolic functions of the gut microbiota may play a significant role in supporting athletes’overall performance in physically demanding activities^19^.

In recent years, the use of integrative omics techniques, such as metabolomics and metagenomics, has revolutionized our understanding of the interactions between the gut microbiome and the host^20^. These tools enable the integration of multiple layers of biological data, facilitating the identification of key metabolic pathways and microbial interaction mechanisms^21–23^. In the context of exercise, it has been proposed that the gut microbiome plays a crucial role in modulating energy systems, both glycolytic and oxidative^24^. For example, short-chain fatty acids (SCFAs), produced by the microbial fermentation of dietary fiber, have been shown to enhance fat oxidation and mitochondrial energy efficiency^25^. On the other hand, gut dysbiosis has been associated with alterations in glucose metabolism, which could affect the efficiency of the glycolytic system during high-intensity exercise^26^. These findings underscore the importance of exploring the interactions between the gut microbiome and energy systems in the context of exercise physiology.

To understand the intricate relationship between sports activities and their effects on the gut microbiome, the utilization of comprehensive multiomics analyses has proven to be a robust investigative toolset^27–30^. By incorporating data from various approaches, such as metagenomics, transcriptomics, proteomics, metabolomics, and lipidomics, it is possible to draw conclusions that provide valuable insights into the molecular mechanisms behind athletic adaptation^19,31^. Among these approaches, metabolomic analysis stands out for its ability to provide a detailed snapshot of the metabolites and their pathways, shedding light on the functional implications of microbiota changes induced by physical activity^32^.

The use of metabolomics in sports fields has grown considerably in recent years because it represents a comprehensive approach for detecting metabolomic changes in response to different physical activity stimuli^33^and because of the advantages that metabolomic methods offer for high-throughput quantification of hundreds of metabolites in a single sample, such as blood, urine, saliva, or feces^29,34^. Moreover, recent research highlights the correlation between the gut microbiota, exercise, and metabolome. Specifically, the presence of the *Butyricicoccus* genus has been linked to higher levels of beneficial HDL cholesterol components and larger HDL particle sizes. Conversely, a decrease in *Ruminococcus *among athletes is correlated with increased total cholesterol and LDL levels. These results suggest that elite athletes exhibit a more favorable lipid profile because the presence of specific gut microbes is associated with better overall health^16^.

This study explores the metabolic profiles (metabolomic and metagenomic signatures at a single time point, rather than longitudinal changes) of elite Colombian athletes—specifically professional cyclists and weightlifters—through a multiomics integration approach guided by knowledge-based strategies. By analyzing metagenomic and metabolomic data, we aim to uncover the relationship between the gut microbiome and host metabolism in these two athletic populations. The inclusion of cyclists and weightlifters enables a comparison of microbiota-associated metabolic patterns linked to oxidative (aerobic) and glycolytic (anaerobic) energy systems. Gaining insight into these metabolic interactions may enhance our understanding of microbiota-driven adaptations across different athletic disciplines.

## Results

### Species and strain taxonomic profiling from samples of Colombian weightlifters and cyclists

**Fig. 1:**
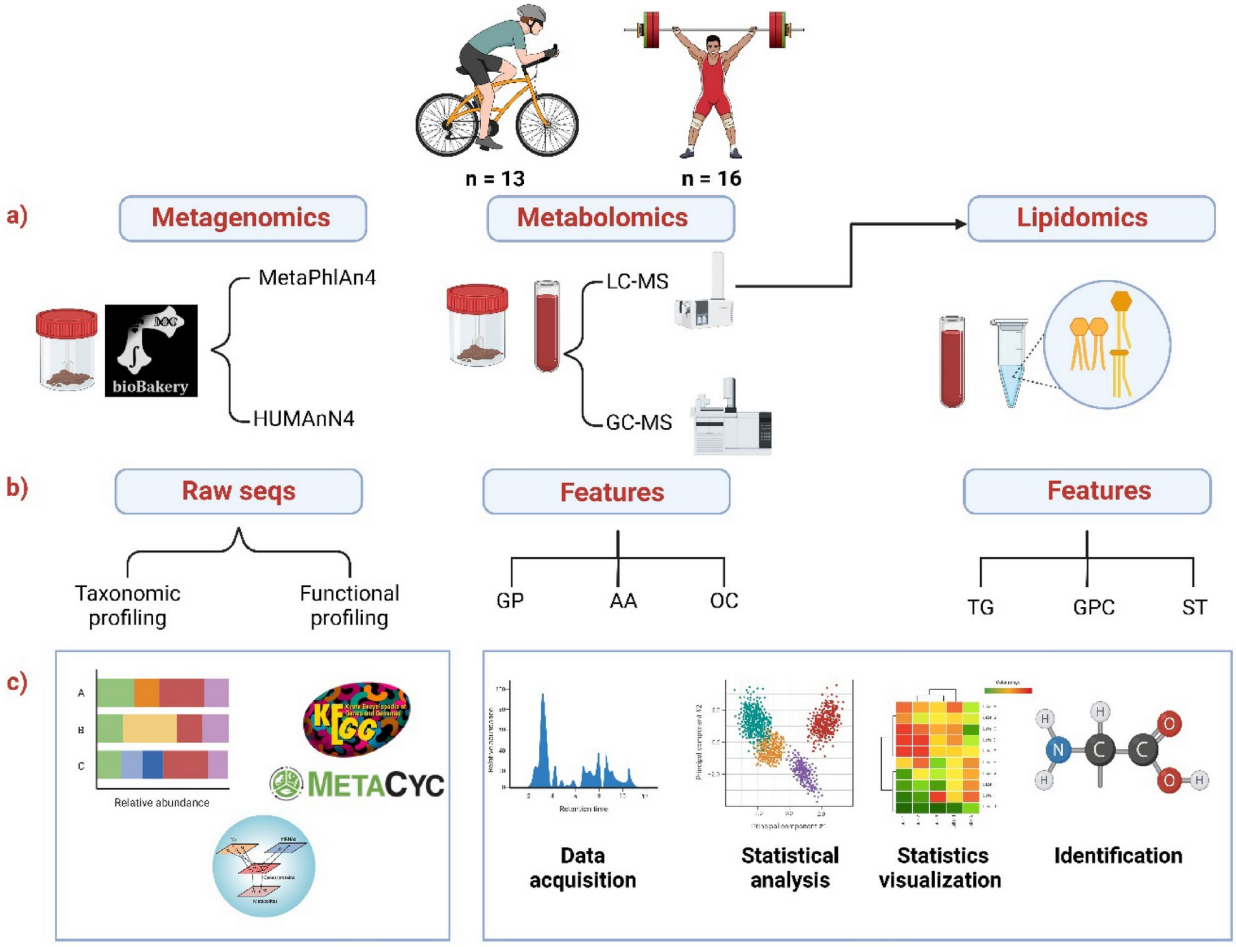
Overview of the integrative omics workflow for studying the gut microbiota and host metabolism in athletes and summarizing the multistep process from sample collection to data integration, providing a holistic view of the experimental approach used to investigate the interplay between the gut microbiota and host metabolism in athletes. (**a**) Sample collection and analytical techniques; this section illustrates the different omics approaches employed in the study. (**b**) Data processing and feature extraction outline the transformation of raw sequences and data into usable features for analysis. In metagenomics, raw sequence data are generated, whereas in metabolomics and lipidomics, feature extraction is performed to identify specific metabolites and lipids from chromatographic data. (**c**) Data integration and analysis showing the integration of different omics data. The artwork was created via Biorender. GP: glycerophospholipid; AA: amino acids; OC: organic compounds; TG: triglycerides; GPC: glycerophosphocholine; ST: sterols. Created in BioRender. Aya, V. (2025) https://BioRender.com/bgs45e5.

In this study, we aimed to characterize the gut microbiota of Colombian elite athletes—weightlifting athletes (WA; n = 16) and cyclist athletes (CA; n = 13)—using a functional analysis approach with the biobakery3 suite, specifically MetaPhlAn4 and StrainPhlAn4 (Fig. 1)^35–37^. This builds upon our previous work, where we conducted a taxonomic profile analysis using Kraken assignment on the same fecal samples, which focused on identifying archaeal and bacterial signatures in these athletes compared with nonathletes^38^. Descriptive data, including age (years: 25.0 ± 8.0 [WA], 27.0 ± 6.0 [CA]), body weight (kg: 62.4 ± 16.1 [WA], 64.0 ± 17.5 [CA]), height (cm: 160.3 ± 10.0 [WA], 164 ± 11.0 [CA]), BMI (kg/m^2^: 24.0 ± 3.8 [WA], 24.9 ± 3.57 [CA]), training frequency (hours/day: 2.0 ± 2.0 [WA], 4.0 ± 0.75 [CA]), training load (days/week: 6.0 ± 0.0 [WA], 6.0 ± 0.5 [CA]), and years as a professional athlete, has been previously published^38^.

For this analysis, we employed the MetaPhlAn4.0 workflow to obtain compositional profiles of the gut microbiota^36^, moving beyond mere taxonomic classification to explore functional potential. We applied a low-abundance filter (10%) and a median abundance value filter, refining the results to approximately 300 species. We then conducted an abundance analysis to identify the most prevalent species across both groups of athletes, highlighting key microbial differences linked to their distinct physical activities. The rationale for comparing cyclists and weightlifters lies in their distinct physiological demands: cyclists primarily rely on oxidative metabolism, whereas weightlifters depend on glycolytic pathways. Given previous findings suggesting a potential link between gut microbiota composition and host energy metabolism, this analysis aimed to identify microbial species and functional differences that may reflect adaptations to these contrasting metabolic requirements.

**Fig. 2:**
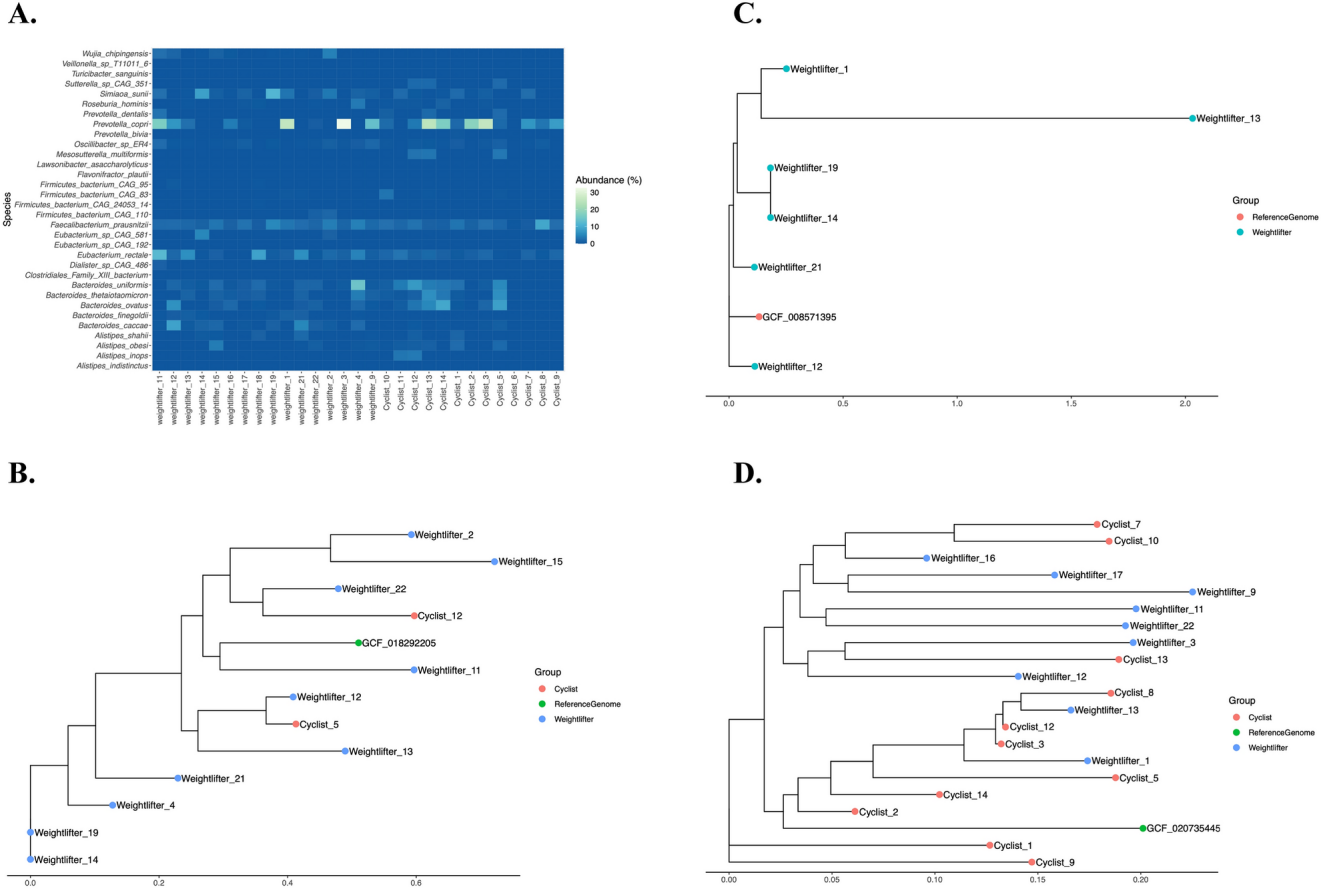
Comparative Analysis of Microbial Species Abundance and Strain Variation in Weightlifting and Cycling Athletes. (**A**) Species level abundance: heatmap of filtered microbial species abundance in weightlifting athletes (WA) and cyclists (CA), with each cell representing the abundance of a species in a sample. Panels **B-D** present the results of StrainPhlAn4 analysis and alignment for the following species: (**B**) *Bacteroides caccae*, (**C**) *Bacteroides finegoldii*, and (**D**) *Prevotella copri*. Figures were created via the ggplot package from RStudio.

Figure 2A visually represents the identified species, and Supplementary Table 2 details them further. The most abundant species identified in both athlete groups included members of the genera *Bacteroides*, *Eubacterium*, *Prevotella*, *Firmicutes*, *Alistipes*, and *Faecalibacterium*.

Statistical analysis was performed to identify important differences between the two groups of athletes: weightlifters (WA) and cyclists (CA). Differential abundance analysis was conducted using MaAsLin2 with a Negative Binomial regression (NEGBIN) model to account for the zero-inflated and overdispersed nature of the microbiome data.

Using NEGBIN, we identified significant differences in the relative abundances of specific bacterial species between cyclists and weightlifters. The most significant species distinguishing between the two athlete groups included *Bacteroides fragilis* (q = 0.03, log2 FC = 2.21), *Alistipes putredinis* (q = 0.04, log2 FC = 1.57), and *Bacteroides uniformis* (q = 0.05, log2 FC = 1.34). In contrast, *Prevotella sp*. CAG_386 (q = 0.08, log2 FC = −2.38) and *Prevotella sp*. CAG_873 (q = 0.09, log2 FC = −1.82) were significantly more abundant in cyclists compared to weightlifters (Supplementary Table 2).

For the weightlifting group (WA), a separate analysis was performed comparing female and male athletes. The results for alpha diversity, using the Shannon index, showed no significant differences between sexes (p-value: 0.64538; FDR: 0.64538). Additionally, a single-factor analysis (Kruskal–Wallis test) revealed no significant features differentiating female and male weightlifters. These findings suggest that, within the weightlifting group, sex-specific differences may not be significant at the microbiome level under the conditions of this study.

Following the initial assessment of microbial community composition, we identified key species of interest for further analysis. Among the species identified, *Bacteroides caccae* (Fig. 2B), *Bacteroides finegoldi I* (Fig. 2C), and *Prevotella copri* (Fig. 2D) emerged as significant due to their prominence in the samples and potential implications for host metabolism. These species were selected for detailed strain-level characterization via StrainPhlAn4^37^. The subsequent distance-based analysis aimed to elucidate the strain diversity and genetic relationships within these species, providing deeper insights into their roles and potential adaptations in the two groups of athletes. As noted in Fig. 2, the analysis produced distance trees, where the X-axis represents the cladistic distance rather than traditional phylogenetic trees.

The sequences from the samples were aligned, and strain trees were constructed, including a reference genome for comparative purposes (supplementary Fig. 1). Figure 2B shows the strain tree built for *Bacteroides caccae*; two distinct clusters corresponding to cyclists and weightlifters are revealed, indicating genetic differentiation between these groups.

Like *B. caccae*, *B. finegoldii* strains from weightlifters formed a tight cluster (Fig. 2C). The short branch lengths in this cluster indicate low genetic diversity, suggesting that the strains are highly similar. The analysis of *P. copri* presented a different pattern (Fig. 2D), with no distinct clustering based on athletic groups. The strains from both cyclists and weightlifters were intermixed, indicating greater genetic diversity within this species.

### Functional profiling of fecal samples from professional athletes

The raw metagenomic data obtained from fecal samples were processed via the HUMAnN4 pipeline^35^. This pipeline allows for the functional profiling of microbial communities by mapping sequence reads to reference databases, such as ChocoPhlAn, and subsequently annotating gene families and pathways. From the HUMAnN4 output, Enzyme Commission (EC) and Kyoto Encyclopedia of Genes and Genomes (KEGG) annotations were extracted for further analysis. The KEGG database and codes were used with permission from Kanehisa Laboratories^39–41^.

**Fig. 3:**
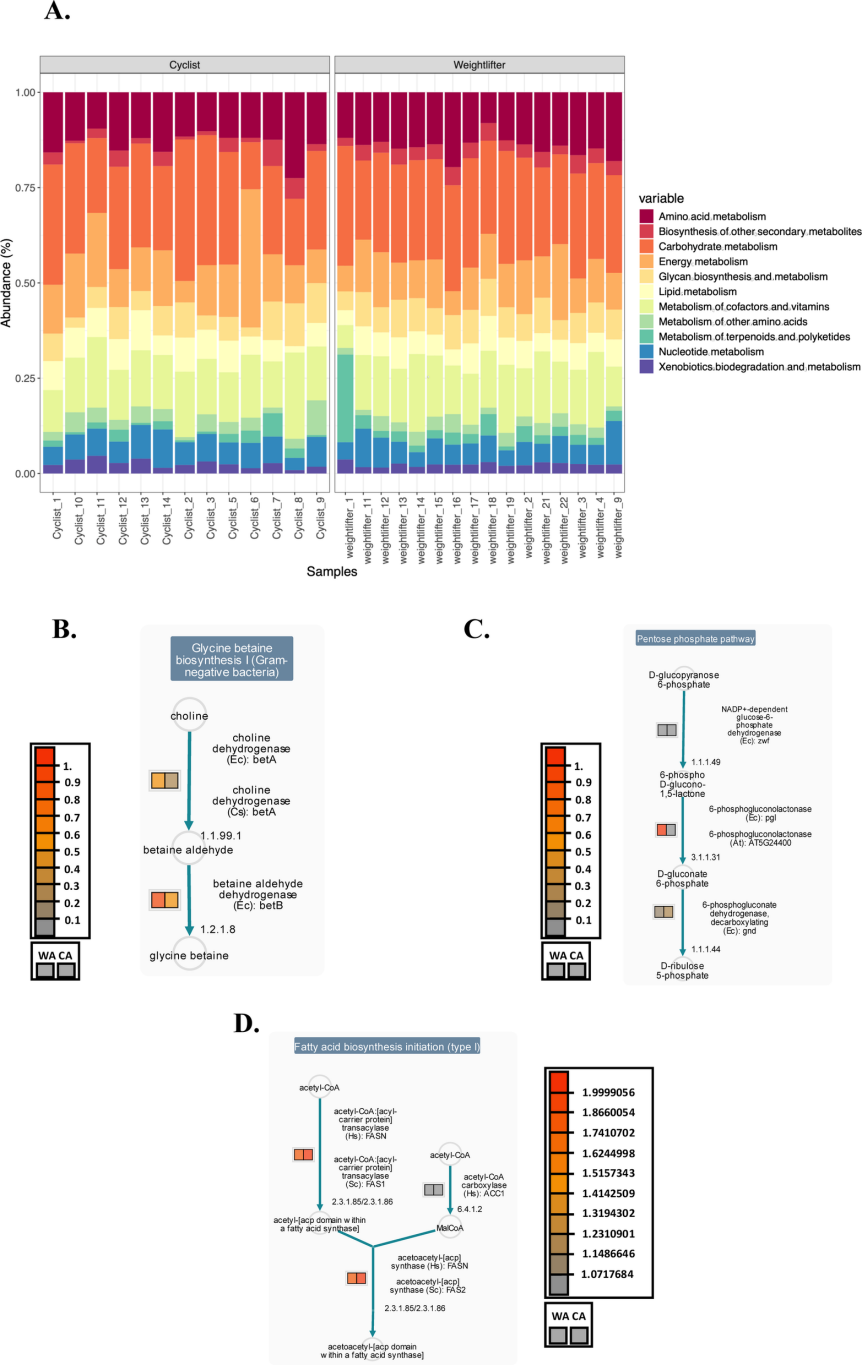
Functional profiling of the gut microbiome of weightlifters and cyclists. (**A**) Functional pathways: Overview of the functional pathways associated with the gut microbiota of professional road cyclists and weightlifters annotated via the Kyoto Encyclopedia of Genes and Genomes (KEGG)^39–41^. While no statistically significant differences were observed, the figure provides a comprehensive visualization of the functional potential of the gut microbiota in both athlete groups. For (**B-D**), MetaCyc analysis was performed via the omics dashboard with Enzyme Commission (EC) codes to generate these pathways. The color scheme in these figures represents the relative abundance of EC in each group: weightlifters (WA) and cyclists (CA). The higher abundance in one group over the other is depicted through gradations of color intensity, from gray to red tones; convention color indicates the major or lower abundance of representative enzymes for each of the pathways selected. (**B**) Pathways related to glycine biosynthesis. (**C**) Representation of the pentose phosphate pathway. (**D**) Fatty acid biosynthesis pathways. (**A**) was created with the ggplot package from RStudio, and (**B–D**) were created with the omics dashboard from the Metacyc server.

Figure 3A provides an overview of the functional pathways associated with the gut microbiota of professional road cyclists (CAs) and weightlifters (WAs), facilitating a comprehensive annotation and interpretation of functional pathways within their gut microbiota. The identified pathways in both groups included amino acid metabolism, carbohydrate metabolism, energy metabolism, glycan biosynthesis and metabolism, lipid metabolism, and the metabolism of cofactors and vitamins.

Analysis with MaAsLin2 did not identify statistically significant differences in enzymatic activity profiles between weightlifters and road cyclists. Given these results, we conducted a secondary analysis using the Omics Dashboard from MetaCyc^42,43^. Features were filtered to exclude ungrouped or unclassified entries, as well as those that did not match any known metabolic pathways in curated databases.

Following this filtering process, enrichment analysis identified distinct enzymatic activity profiles in the gut microbiota of weightlifters compared with those of road cyclists. Specifically, significant enrichment was observed in pathways such as L-arginine biosynthesis III (via N-acetyl-L-citrulline), fatty acid biosynthesis initiation (type II), streptorubin B biosynthesis, methylerythritol phosphate pathways I and II, GDP-mannose biosynthesis, pectin degradation I, glycine degradation, and 4-deoxy-L-threo-hex-4-enopyranuronate degradation. Notably, the bacteria *Bacteroides finegoldii* was highly represented, indicating its significant role in the detected enzymatic reactions (Supplementary Table 3, supplementary Fig. 5).

To explore these findings comprehensively, Metacyc’s omics system was utilized^42,43^. which facilitates the integration of metagenomic data in EC format, allowing for the visualization and interpretation of metabolic pathways and enzymatic reactions across datasets. The resulting integrated analysis provided a detailed metabolic pathway map highlighting distinctions between cyclists and weightlifters (Figs. 3B-D, supplementary Fig. 5). It is important to note that while the differences observed in these pathways demonstrated a trend toward significance (FDR = 0.1236), they do not meet the commonly used threshold of FDR < 0.05.

This analysis revealed that the biosynthesis of amino acids was more prominent in weightlifters, whereas cyclists exhibited increased biosynthesis of fatty acids. Additionally, substrate degradation pathways, such as those involving carbohydrates and amino acids, were more active in weightlifters than in cyclists. A subtle difference was noted in the degradation of fatty acids in cyclists. Both groups showed fermentation to short-chain fatty acids, whereas cyclists exhibited pathways related to methanogenesis and weighted the pentose phosphate pathway. In particular, the weightlifter gut microbiome contains representative enzymes that reflect the presence of pentose phosphate pathways (Fig. 3C).

Supplementary Table 3 lists relevant enzymatic reactions, associated pathways, and EC code numbers obtained from HUMAnN4.0 analysis in Colombian athletes (WA and CA).

### Metabolomic analysis of two different sport activities

To gain deeper insights into potential distinctions between athletes predominantly using glycolytic pathways (such as weightlifters) and those favoring oxidative pathways (such as road cyclists), we conducted an integrative omics analysis. This comprehensive approach included examination of the fecal metabolome, as well as plasma metabolomic and lipidomic analyses.

#### Metabolomic analysis of fecal samples

First, metabolomic analysis of fecal samples from WAs (n = 16) and CAs (n = 13) was performed via liquid chromatography‒mass spectrometry (LC‒MS) and gas chromatography‒mass spectrometry (GC‒MS) platforms. The data were subjected to diverse procedures, as detailed in the methods section and Fig. 1. Before the annotation process^44^, platform stability was confirmed by performing quality control (QC) assessments, ensuring consistent performance across all platforms. The clustering of the QC samples is shown in Supplementary Fig. 2. Next, we obtained the set of metabolites listed in supplementary Table 4. Statistical analysis was performed; nevertheless, multivariate models did not reveal different profiles between groups, and univariate analysis revealed diverse metabolites with significant abundances (p value, FDR ≤ 0.05). A further gender-based reanalysis of the fecal samples in the weightlifting group was performed. Both two-sample t-tests and Wilcoxon rank-sum tests, using a p-value and FDR cutoff of 0.05, revealed no significant differences between male and female athletes.

**Fig. 4:**
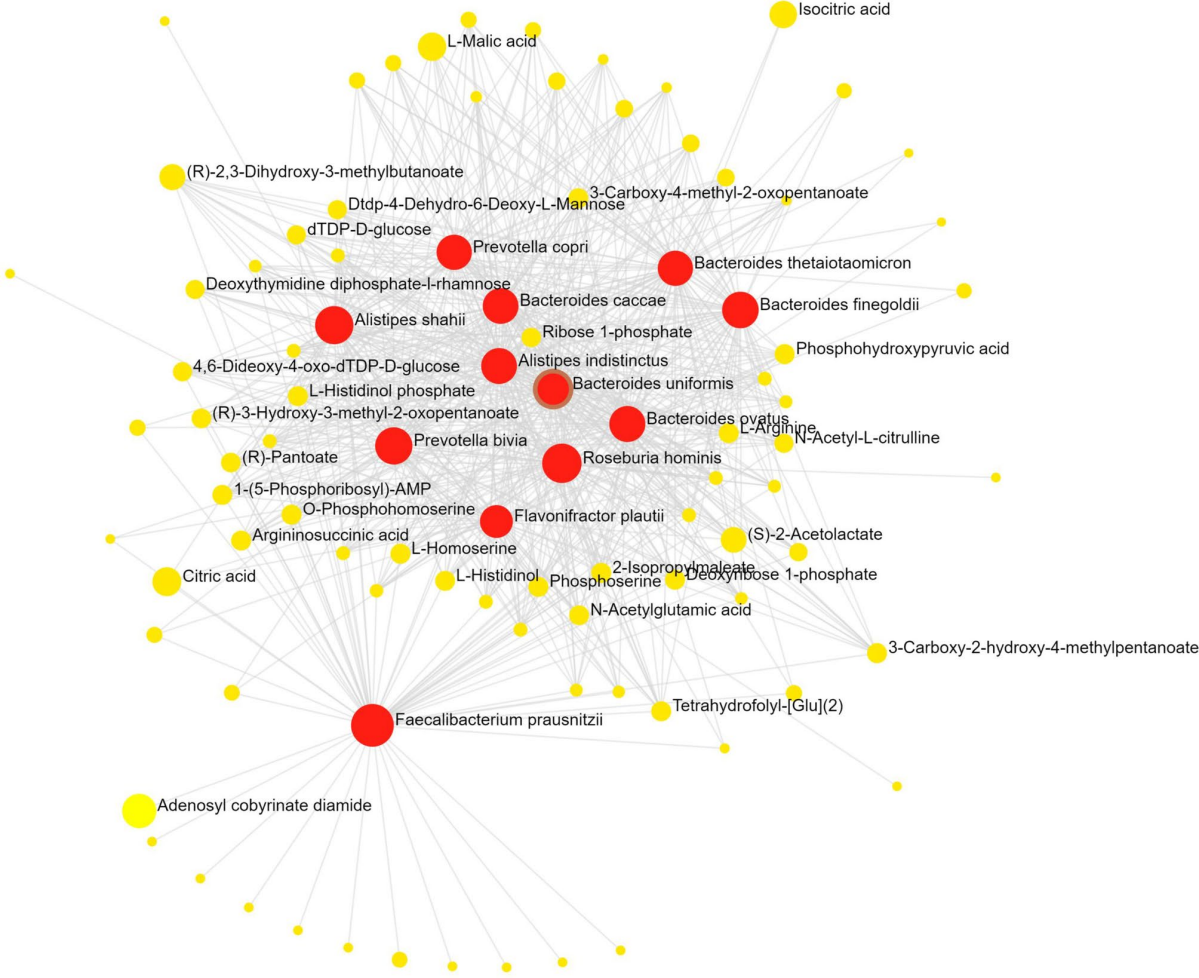
Fecal metabolomics and integrative analysis. Network analysis of microbial species and metabolites: This network diagram illustrates the connections between key microbial species and their associated metabolites. Red dots represent microbial species, yellow dots denote metabolites, and the lines indicate significant correlations between them^45^. The prediction of metabolic potential for different microbial taxa was conducted via the OmicsNet web server^45^, which employs logistic regression models based on high-quality genome-scale metabolic models (GEMs). This tool enables further enrichment of the network by integrating protein‒metabolite interactions to identify potential enzymes, as shown in Table 1.

Figure 4 shows that the results from the t-test analysis revealed metabolites such as O-behenoyl carnitine, hydroxy-gamma-tocotrienol, docosadienoyl carnitine, beta-alanine betaine, arachidyl carnitine, and LPA 21:0, indicating statistically significant differences in their abundances between the two groups of athletic disciplines, with a greater abundance of fatty acyls in the weightlifter group. Next, we investigated the possible metabolites produced by the gut microbiota. The reported metabolites, such as glycolate, hydroxy butyric acid, nicotinate, beta-alanine betaine, ethanolamine, L-valine, dopamine, histidinal, and hyodeoxycholate, among others, were classified as theoretically produced by the gut microbiota.

**Table 1:** Pathway enrichment analysis of metagenomic data from the gut microbiomes of Colombian weightlifters and road cyclists.

Pathway	FDR	P-Value
Phenylalanine, tyrosine and tryptophan biosynthesis	4.53E-07	3.31E-09
Arginine biosynthesis	4.53E-07	3.73E-09
Valine, leucine and isoleucine biosynthesis	4.53E-07	3.73E-09
Histidine metabolism	7.04E-05	7.71E-07
Folate biosynthesis	0.000216	2.96E-06
Citrate cycle (TCA cycle)	0.0387	0.00138
Biosynthesis of secondary metabolites—other antibiotics	0.0523	0.00201
Glycine, serine and threonine metabolism	0.057	0.00234
Glucagon signaling pathway	0.0684	0.003
Pentose phosphate pathway	0.122	0.00702
Glycerophospholipid metabolism	0.29	0.0207
Glycosylphosphatidylinositol (GPI)-anchor biosynthesis	0.383	0.0336
Vitamin B6 metabolism	0.424	0.0395

FDR: False discovery.

After these metabolites were identified, a knowledge-driven omics analysis was performed with the species identified through MetaPhlAn4 (Fig. 2A) and the subset of metabolites produced by the gut microbiota. This integrative approach utilized OmicsNet 2.0^38^ to explore potential interactions between microbial species and metabolites (Fig. 4)^46^.

The resulting network highlighted key microbial species and their associated metabolites (Fig. 4), providing insights into how specific gut microbiota might contribute to the metabolic profiles observed in weightlifters and road cyclists. This approach facilitated a comprehensive understanding of the potential biological mechanisms underlying the differences in metabolite production and metabolic pathways between the two groups of athletes.

Using OmicsNet, we mapped interactions and identified key nodes in the network for potential further investigation. This integrative analysis emphasizes the value of considering both microbial and metabolomic data to elucidate the complex relationships among physical activity, the gut microbiota, and host metabolism. In this model, the yellow dots represent metabolites hypothetically produced by the gut microbiota and identified through fecal metabolomic analysis, whereas the red dots indicate microbial species identified via the MetaPhlAn4.0 pipeline (Fig. 2A). The OmicsNet analysis revealed enriched metabolic pathways, which are graphically represented by the nodes in Fig. 4. The pathways showing significant correlations between species and metabolites included phenylalanine, tyrosine, and tryptophan biosynthesis; arginine biosynthesis; valine, leucine, and isoleucine biosynthesis; histidine metabolism; folate biosynthesis; and vitamin B6 metabolism, as detailed in Table 1.

### Plasma metabolome and lipidome of Colombian athletes with different energy demands

With the aim of identifying metabolic differences within the blood matrix of athletes, with a specific focus on reflecting potential variations between two distinct groups: athletes predominantly utilizing glycolytic pathways (weightlifters) and those primarily relying on oxidative metabolism (cyclists), metabolomic and lipidomic approaches were employed.

**Fig. 5:**
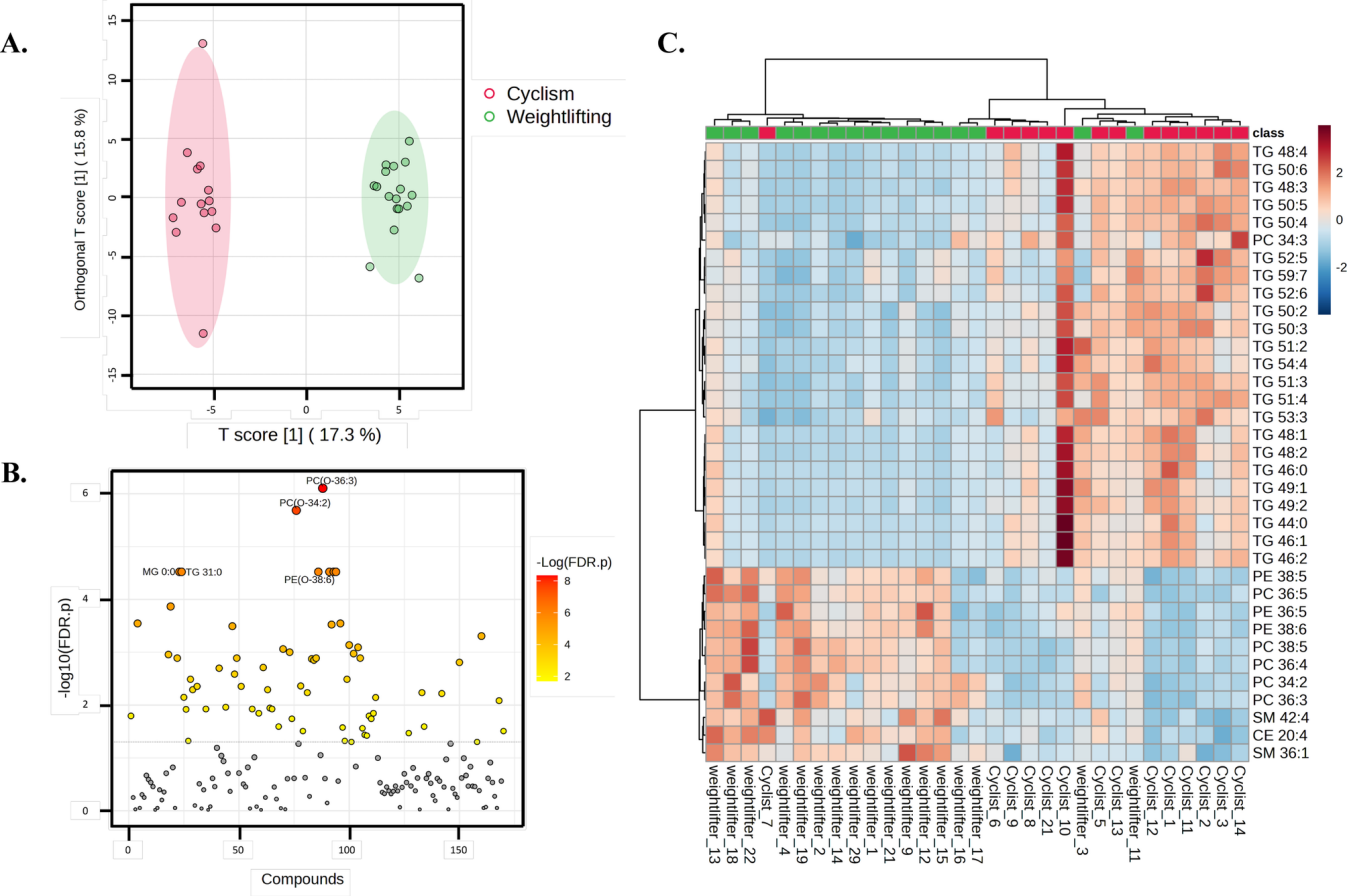
Plasma metabolome and lipidome of Colombian athletes with different energy demands. (**A**) Orthogonal partial least squares discriminant analysis (OPLS-DA) reveals significant separation between the sample groups, with notable performance metrics. (**B**) T-test analysis was conducted to visualize the differences in metabolite abundance between the two types of sports (WA—weightlifters and CA—cyclists). The p-value threshold for statistical significance was set at 0.05 FDR, as indicated in the (**C**) Hierarchical clustering heatmap from lipidomic analysis: This analysis provides a comprehensive overview of the lipidomic species identified across the blood samples of the WA and CA groups. Notably, triglycerides have a strong abundance profile in cyclists, whereas other types of lipids, such as sterols and phosphocholines, are more abundant in weightlifters. Graphics and comprehensive analysis were performed with MetaboAnalyst 6.0^48^.

Through the utilization of metabolomic techniques, this assay aimed to provide a comprehensive profile of the small molecule metabolites found in athletes’plasma blood samples. Liquid chromatography coupled with tandem mass spectrometry (LC‒MS/MS) and gas chromatography coupled with tandem mass spectrometry (GC‒MS/MS) were employed for the analysis of the plasma metabolome from WAs (n = 16) and CAs (n = 13). Following the annotation process, approximately 120 compounds were identified, and subsequently, a reduction analysis was performed via linear modeling^47^. The resulting metabolites from this analysis are highlighted in supplementary Table 5 and are visualized in Fig. 5B.

Please replace for the one attached due to low resolutionDifferent classes of metabolites, including carboxylic acids, carbohydrates, dicarboxylic acids, fatty acids, fatty acyls, indoles, organic carbonic acids, organonitrogen compounds, sphingolipids, steroids, sterols, and tricarboxylic acids, among others, were detected (Supplementary Table 5). Among the identified compounds, glycerophosphocholines and glycerophospholipids were the most abundant. Significant differences were observed in families such as glycerophosphoethanolamines, medium-chain fatty acids, monoradylglycerols, steryl esters, and dicarboxylic acids, with greater abundance in plasma samples from the WA group than in those from the CA group (p ≤ 0.05; FDR ≤ 0.05). In contrast, metabolites with higher abundances in the CA group were predominantly from the subclass triradylglycerols.

In addition to multivariate analysis (Fig. 5A), univariate analysis was conducted to identify significant differences between groups (Fig. 5B).

The multivariate models (MVAs) demonstrated successful group separation, as evidenced by the distinct clustering in the score plots. Model quality was evaluated via R^2^ and Q^2^ values, confirming strong predictive power and explained variance. Specifically, the model accounted for 45.4% of the cumulative variation in X (metabolite data) and 98.9% in Y (group), with a high cumulative predictive ability of 76.6% and a significant p value of 2.0283e-05. These results emphasize the effectiveness of the OPLS-DA model in distinguishing between different metabolic profiles, highlighting the importance of the observed differences. Additionally, univariate analysis (UVA) was performed to further explore the significance of individual metabolites, complementing the findings from the MVA.

Secondary gender-based analyses were conducted as part of the reanalysis of plasma metabolomics and lipidomics data, alongside the overall analysis. Two-sample t-tests and Wilcoxon rank-sum tests were performed, and while significant features were identified with a p-value of 0.05, the False Discovery Rate (FDR) adjustment did not yield any significant results.

To further identify significant differences between groups, fold changes and t tests were applied (Supplementary Table 5). Figure 5B displays major differences found in the weightlifter group (WA), with increased levels of monoacylglycerol (MG 18:0), triglyceride (TG 31:0), medium-chain fatty acids (FA 18:2), lysophosphatidylcholine (LPC 18:3), caprylic acid, and diacylglycerol (DG 27:0).

To provide a comprehensive overview of the top 25 features identified through the plasma metabolomic analysis, a heatmap was constructed. The selection was based on significant changes observed, with a p value and FDR threshold set to ≤ 0.05, and MetaboAnalyst 6.0^48^ was used to prioritize these compounds. This approach allowed us to focus on the most relevant features, which are visually represented in Supplementary Fig. 3, highlighting key metabolic signatures related to the WA and CA groups.

### Lipidomic analysis

Multivariate and univariate statistical analyses, such as principal component analysis (PCA), partial least squares discriminant analysis (PLS-DA), and t tests, were used to identify differential lipid species and elucidate metabolic patterns associated with experimental conditions. The orthogonal partial least squares discriminant analysis revealed robust separation between sample groups on the basis of their plasma lipidomic profiles. This model exhibited high performance metrics, R^2^X(cum): 0.626 R^2^Y(cum): 0.984 Q^2^(cum): 0.808, and a significant p value (3.14953e-05) (Supplementary Fig. 4A).

Plasma lipidomic analysis revealed distinct lipid profiles between the experimental groups, with significant differences observed in the abundance of specific lipid species. The lipid species demonstrating the most notable disparities in abundance were identified, namely, triglycerides (TGs) and phosphatidylcholine (PC).

As shown in Fig. 5C and supplementary Fig. 4B, triglycerides (TGs) were significantly elevated in cyclists compared with weightlifters. This observation suggests a heightened reliance on lipid metabolism and energy utilization during endurance exercise. TGs serve as essential energy sources during prolonged aerobic activities, such as cycling, where sustained effort demands efficient lipid oxidation for ATP production. The specific TG species identified, including TG54:6, TG52:4, and TG54:3, may reflect adaptations to endurance training, facilitating enhanced lipid mobilization and utilization to sustain energy production over extended periods. Elevated levels of TGs with shorter carbon chains, such as TG50:2 and TG50:1, may indicate increased lipolysis and fatty acid turnover, supporting energy production during endurance exercise bouts. An enrichment analysis performed with LION/web^49,50^ highlighted the upregulated pathways involved in lipid droplet formation, lipid storage, and glycolipid synthesis, among other processes (supplementary Table 6, supplementary Fig. 4B-C).

Phosphatidylcholine (PC) and phosphatidylethanolamine (PE) species are notably enriched in weightlifters compared with cyclists. PC and PE are essential components of cell membranes and play critical roles in membrane integrity, signaling, and lipid metabolism (Supplementary Fig. 4C).

## Discussion

Our study aimed to explore the intricate metabolic patterns associated with weightlifting and road cycling. Using metagenomic, metabolomic, and lipidomic profiling, (Fig. 1); we investigated potential pathways linking athletes’gut microbiomes to their systemic metabolism. These findings contribute to the growing body of evidence suggesting that the gut microbiota of athletes may undergo metabolic adjustments to support energy metabolism and bolster physiological resilience during physical activity^10,19,24,38,50–54^. Future studies should explore how these microbial changes contribute to metabolic adaptations in different types of athletes, providing insights into potential applications in sports science. This could ultimately contribute to the development of individualized training and nutritional strategies that enhance athletic performance.

Despite not reporting robust differences between the taxonomic and functional microbiotas of both groups (WA—CA), as seen in the multivariate and univariate analyses (see Supplementary Table 2), our MetaPhlAn4.0-incorporated analysis provides valuable insight into the gut microbiota of Colombian athletes, as shown in Fig. 2A and Supplementary Table 1. The most abundant species identified across both groups included *Bacteroides*, *Eubacterium*, *Prevotella*, *Alistipes*, and *Faecalibacterium*. These genera are well-documented for their roles in various metabolic processes^55^, and their relative abundances may reflect adaptations to the specific physiological demands of endurance and strength-based sports^24,33,56^. These findings are in concordance with other related research, where gut microbiota members are recognized for their metabolic benefits to the host^9,57–60^.

The relevance of *Bacteroides *species in the gut microbiota of athletes is underscored by their ability to break down a wide range of dietary polysaccharides through carbohydrate-active enzymes (CAZymes)^61^, producing metabolites such as acetate, propionate, and succinate, which play crucial roles in maintaining gut mucosal integrity and immune defenses^62^. Additionally, *Bacteroides *species exhibit diversity at the species and strain levels in their production of short-chain fatty acids, which is influenced by factors such as diet and physical activity^24,56,63^. This metabolic flexibility may contribute to their prevalence in athletes, where different physical activities demand specific metabolic adaptations^56^. Moreover, *Bacteroides *are involved in the modification of bile acids, impacting host metabolism and immune function, which could further explain their significant presence in athletes^54^. Further analysis using StrainPhlAn4 identified two specific *Bacteroides* strains, *Bacteroides caccae* (Fig. 2B) and *Bacteroides finegoldii* (Fig. 2C), which were associated with weightlifters and road cyclists. *B. caccae*has been previously linked to sports with highly dynamic and static components, such as rowing^2^, suggesting its role in supporting the physical demands of these activities.

Although *B. finegoldii *has not been directly associated with sports, it has been identified in highly active military subjects, hinting at its potential relevance to physically demanding lifestyles^56^. Moreover, we identified *Bacteroides thetaiotaomicron* (Supplementary Table 2, Fig. 2A), which is known for its ability to break down complex carbohydrates and increase nutrient absorption and energy availability, which is particularly beneficial for athletes who require efficient energy metabolism during training and competition. Research also indicates that *B. thetaiotaomicron *is present in the gut microbiota of sports players^64^, suggesting its potential role in maintaining gut health in competitive environments. Its presence may contribute to the overall metabolic health of athletes, supporting their performance through improved digestion and nutrient utilization. Similarly, *Bacteroides uniformis *plays a crucial role in carbohydrate fermentation and is associated with the production of SCFAs, which are beneficial for gut health and may influence inflammation and recovery in athletes^53^. Studies have shown that *B. uniformis *is often found in the gut microbiota of physically active individuals, including athletes, where its presence is linked to a balanced gut microbiome^54^.

Similar to the *Bacteroides* genus, we identified diverse *Prevotella* species in our analysis, including *Prevotella copri*, *Prevotella stercorea*, *Prevotella bivia*, and *Prevotella dentalis*. Although no significant differences were detected between the athlete groups, these results align with previous reports that established *Prevotella *as a prominent member of the gut microbiota in cyclists, particularly in North America^65^. *Prevotella* species, especially *P. copri*, are known for their involvement in the metabolism of complex carbohydrates. As shown in Fig. 2A, *Prevotella copri *stands out for its relatively high abundance across samples, emphasizing its potential role in the gut microbiota of athletes. They play a crucial role in fermenting dietary fibers into short SCFAs, which can be utilized as an energy source during prolonged physical activity^66^. This metabolic capability is particularly advantageous for endurance athletes, such as cyclists, who rely on sustained energy release during extended periods of exercise^38^.

The report of specific microbial species between weightlifters and cyclists further underscores the potential role of the gut microbiome in athlete performance and health. For instance, the higher abundance of *Prevotella *in cyclists has been linked to enhanced carbohydrate metabolism and SCFA production^65^, which could support endurance performance by improving energy availability and reducing inflammation. Similarly, the enrichment of *Bacteroides *in weightlifters may reflect a microbiome adapted to high-protein diets^34^, as these species are known to thrive on protein-derived substrates and contribute to nitrogen cycling and amino acid availability. The presence of *Akkermansia muciniphila* in both groups, but particularly in cyclists, is noteworthy due to its association with improved metabolic health, gut barrier function, and immune regulation. These properties could enhance recovery and reduce the risk of overtraining syndrome in endurance athletes.

While taxonomic differences were subtle, we further explored functional microbial activity to assess potential distinctions in metabolic pathways between weightlifters and cyclists. This approach allowed us to investigate whether metabolic function, rather than species composition, differentiates athlete microbiomes. Through HUMAnN4 analysis, we identified numerous carbohydrate degradation enzymes from the gut microbiota family members. Although no significant differences were observed, the MetaCyc omics dashboard revealed the presence of multiple pathways involved in polysaccharide degradation and carboxylic acid degradation. Additional details regarding these pathways are provided in Supplementary Fig. 5^43^. Fecal metabolomic assays further allowed us to identify key metabolites, such as oxalate, propanoate, succinate, and glycolate, among others (Fig. 4 and Supplementary Table 4), reflecting the metabolic activities of the gut microbiota and their potential contributions to athlete performance. To further elucidate these metabolic processes and gain a more comprehensive understanding, incorporating additional omics approaches, such as RNA sequencing^67^and single-cell metabolomics^68^, could be invaluable. These advanced techniques would allow the exploration of gene expression patterns and metabolic activity at a more specific level, providing deeper insights into the functional roles of the gut microbiota in athletic performance and how these may vary between individuals or under different environmental conditions.

Our integrative omics analysis further revealed that the enriched pathways identified via the integration of metagenomic, and metabolic data were significantly related (p < 0.005) to the biosynthesis of phenylalanine, tyrosine, and tryptophan, as well as the biosynthesis of arginine, valine, leucine, and isoleucine (Table 1). These pathways are critical for protein synthesis and metabolic functions that support athletic performance. The functional analysis of enzymes also revealed a significant presence of enzymes encoding amino acid synthesis, which complements our fecal and plasma metabolome analysis that identified the presence of L-valine, a key BCAA (Fig. 4).

The integrative analysis performed in this study provided significant insights into the metabolic functions of key microbial genera, as illustrated in Fig. 4 and Table 1. These microbial activities are essential for energy production, muscle recovery, and overall athletic performance (Fig. 3B-D). On the basis of these findings, our lipidomic analysis further elucidated the metabolic landscape in athletes (Fig. 5C). Examination of the metagenome and metabolome data revealed a prominent presence of lipids and lipid-like substances across both groups of athletes (see Fig. 4 and Fig. 5). Specifically, our analysis of fecal metabolites revealed significant quantities of various lipid classes, including fatty acyl carnitines, amino acid derivatives, steroids, phosphatidylcholines (PCs), and phosphatidylethanolamines (PEs), as shown in supplementary Tables 2 and 4. These lipid metabolites are critical for energy metabolism, cellular signaling, and membrane integrity, all of which are vital for maintaining high levels of physical performance^29,34,69,70^. Enrichment analysis of the lipidome results revealed upregulated pathways linked to lipid droplet formation, lipid storage, and glycolipid synthesis (Supplementary Table 6, Supplementary Fig. 4B-C), emphasizing the importance of these processes in enhancing athletic performance.

**Fig. 6:**
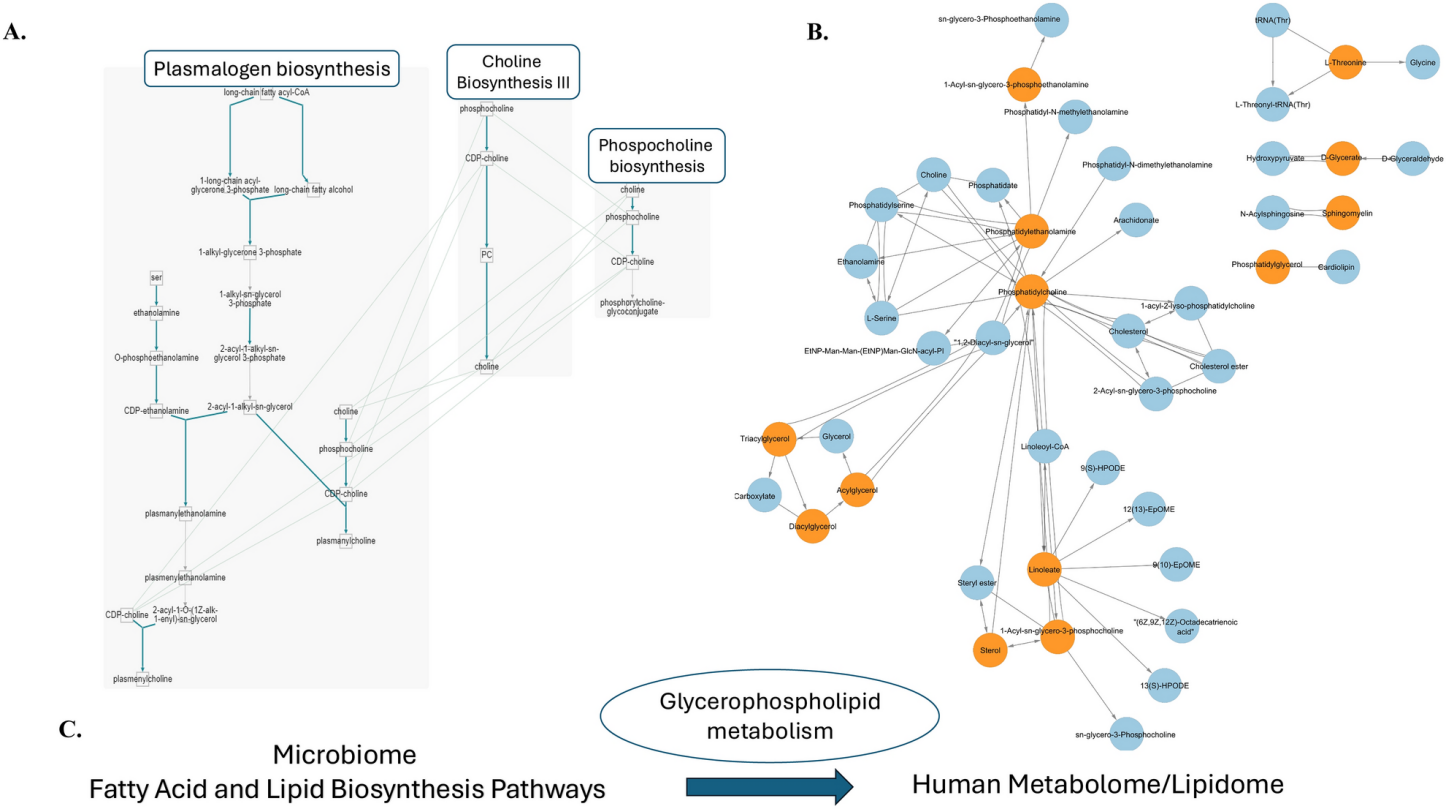
Hypothetical Framework Illustrating Potential Interactions between Gut Microbiome Metabolic Reactions and Key Metabolite Classes and Pathways. This figure presents a schematic representation illustrating potential links between gut microbiome reactions identified through metagenomics analysis and key metabolite classes along with their associated pathways. (**A**) Shows specific biosynthesis pathways, including Plasmalogen biosynthesis, Choline biosynthesis III, and Phosphocholine biosynthesis. Pathways were built with representative enzymes from metagenomic data^43^. Graphic 6 A was created through the pathway analysis tool from Metacyc’s server. (**B**) depicts a network of metabolites interconnected by their potential associations, with glycerophospholipid metabolism highlighted as a central theme. Network B was constructed via Cytoscape 3.0 software^82^. Significant metabolites and lipids identified from the metabolomic and lipidomic data are represented by orange circles, while the enriched pathways are shown in blue circles. (**C**) Illustrates the broader context, showing the interaction between microbiome fatty acid and lipid biosynthesis pathways and the human metabolome/lipidome. The connections depicted in this figure are hypothetical and serve as a conceptual framework for understanding the interplay between gut microbial activity and human metabolite/lipid production.

To explore these interactions further, Fig. 6 presents a schematic representation that hypothesizes potential links between gut microbiome metabolism and the host metabolome/lipidome. This figure offers a conceptual framework for understanding the interplay between gut microbial activity and metabolite production. However, these connections are hypothetical and require further investigation to fully map and validate the comprehensive metabolic relationships involved. Previous studies have shown that athletes exhibit increased levels of fecal metabolites such as SCFAs such as butyrate, acetate, propionate^64,71^, ammonia^72^, and amino acids and their derivatives. Although we did not describe SCFAs in detail in our current study, we identified 3-hydroxybutanoate (see supplementary Table 4 and Fig. 4) in the fecal metabolome and detected related pathways in MetaCyc´s dashboard^43^. This metabolite, found in both fecal and plasma samples from weightlifters and cyclists, suggests a significant role in the metabolic processes of these athletes, particularly concerning fatty acid and lipid biosynthesis/degradation and cofactor synthesis. Subpathways related to phosphatidylcholine synthesis, phosphatidylcholine resynthesis via glycerophosphocholine, and plasmalogens were also identified (Fig. 6).

Our analysis identified differences in microbial metabolic pathways between weightlifters and cyclists, particularly those related to energy metabolism, amino acid biosynthesis. These pathways have direct implications for athlete performance and recovery. For example, the enrichment of pathways such as glycolysis, the TCA cycle, and oxidative phosphorylation in cyclists suggests a microbial community optimized for sustained energy production, which aligns with the aerobic demands of endurance sports. In contrast, weightlifters exhibited higher abundances of pathways associated with anaerobic metabolism, such as lactate fermentation, which may support the rapid energy demands of high-intensity, short-duration activities.

The increased abundance of pathways involved in branched-chain amino acid (BCAA) biosynthesis in weightlifters may support muscle protein synthesis and recovery, which are critical for strength training adaptations. On the other hand, the higher levels of aromatic amino acid biosynthesis pathways in cyclists could play a role in neurotransmitter production, potentially influencing fatigue resistance and mental focus during prolonged exercise. Additionally, the higher abundance of SCFA-producing pathways in cyclists, particularly butyrate and propionate, may enhance gut barrier integrity, reduce systemic inflammation, and improve energy harvesting from dietary fiber. These effects could contribute to better recovery and overall health in endurance athletes.

Our study hypothesized that weightlifting and cycling, which involve different energy systems, are associated with distinct gut microbiome compositions and functional profiles. However, our metagenomic and fecal metabolome analyses did not reveal clear differences between these sports. Despite this, our plasma metabolome and lipidome analyses yielded significant findings. The multivariate analysis of the plasma metabolomic and lipidomic data revealed meaningful differences that were not apparent in the metagenomic and fecal analyses.

The higher abundance of PCO-34:2, PC18:0, PEO-36:5, and PEO-38:5 in weightlifters suggests that distinct lipid metabolic adaptations are associated with resistance training and muscle adaptation. These lipid species may contribute to membrane remodeling processes and cellular adaptations to mechanical stress and metabolic demands characteristic of weightlifting activities. Furthermore, the presence of specific PC and PE species, such as PCO-36:5 and PEO-38:6, may reflect unique lipidomic signatures associated with muscle tissue remodeling and repair processes following intense resistance exercise.

This discrepancy highlights the importance of using a multiomic approach to gain a comprehensive understanding of the metabolic adaptations associated with different types of physical activity. Our results suggest that while the gut microbiome may not vary significantly between weightlifters and cyclists in terms of composition or function, the systemic metabolic responses observed through plasma and lipidomic analyses provide valuable insights into how these athletes adapt to their specific training regimens.

The observed differences in microbial composition and metabolic pathways suggest that the gut microbiome may play a key role in modulating athlete performance and health. The microbiome’s ability to influence energy metabolism, substrate utilization, and neurotransmitter production could directly impact athletic performance^10^. For example, lipid biosynthesis in cyclists may improve endurance capacity by enhancing fat oxidation and reducing glycogen depletion. Similarly, the microbiome’s role in modulating inflammation, oxidative stress, and muscle protein synthesis could influence recovery processes. The enrichment of BCAA biosynthesis pathways in weightlifters may accelerate muscle repair and adaptation following resistance training. Furthermore, the gut microbiome’s impact on immune function, gut barrier integrity, and systemic inflammation could affect overall athlete health and resilience. A balanced microbiome may reduce the risk of infections, gastrointestinal distress, and overtraining syndrome, which are common concerns among athletes^58,72^.

In conclusion, this study explored the intricate relationship between the gut microbiota and metabolic adaptations in athletes via diverse omics approaches. Key microbial genera such as *Bacteroides* and *Prevotella* were found to be integral to metabolic functions essential for elite athletes, influencing energy metabolism, muscle recovery, and performance. The incorporation of lipidomic data enhanced our understanding of the role of the gut microbiota in lipid metabolism and cellular functions crucial for athletes. Although not all variables were addressed, this work provides a foundational framework for future research on how the gut microbiota and metabolic profiles affect sports performance. These findings emphasize the complex interplay between the gut microbiota, metabolic pathways, and athletic outcomes, advancing our understanding of microbiome changes associated with different training regimens.

### Future directions

While our findings highlight the potential functional implications of the gut microbiome for athlete performance and health, further research is needed to establish causal relationships and explore therapeutic interventions. For example, targeted dietary strategies, probiotics, or fecal microbiota transplantation could be investigated as tools to optimize the gut microbiome for specific athletic demands. The findings of this study open new avenues for translating microbiome research into practical applications for athlete performance, recovery, and health. One promising direction is the development of personalized nutrition strategies tailored to the specific demands of different athletic disciplines. For instance, weightlifters, who exhibited microbial communities enriched in pathways related to amino acid biosynthesis, might benefit from diets that support these metabolic functions, such as increased intake of high-quality proteins and specific fibers that promote microbial diversity. On the other hand, cyclists, whose microbiomes showed a higher capacity for lipid biosynthesis and energy metabolism, could optimize their performance through diets rich in complex carbohydrates and healthy fats, which may further enhance these microbial functions.

Another potential application lies in the design of targeted probiotic interventions. The identification of key microbial taxa, such as *Prevotella* and *Bacteroides*, which were differentially abundant between weightlifters and cyclists, suggests that these bacteria could serve as biomarkers for microbiome-based interventions. For example, probiotics containing *Prevotella* strains might be explored for their potential to improve endurance capacity by enhancing lipid biosynthesis and energy harvesting, while *Bacteroides*-based formulations could support muscle recovery and nitrogen cycling in strength athletes.

To build on the current findings, future studies should address specific research questions and employ robust methodologies. For example, longitudinal studies could investigate how changes in diet, training intensity, or competition schedules influence the gut microbiome and its functional capabilities. Integrating multi-omics approaches, such as metagenomics, metabolomics, and proteomics, would provide a more comprehensive understanding of the interactions between microbial metabolism and host physiology. Additionally, intervention studies could evaluate the efficacy of personalized nutrition plans or probiotic supplements in modulating the microbiome and improving athletic outcomes. These studies should also consider the impact of individual factors, such as genetics, lifestyle, and baseline microbiome composition, to develop more precise and effective interventions.

Finally, the potential applications of these findings extend beyond elite athletes to recreational exercisers and individuals seeking to improve their physical fitness. By elucidating the links between the gut microbiome, lipid biosynthesis, and energy metabolism, this research could inform broader strategies for enhancing exercise performance, recovery, and overall metabolic health in diverse populations. Collaborative efforts between microbiologists, nutritionists, and sports scientists will be essential to translate these insights into practical tools and guidelines for optimizing human health through microbiome modulation.

### Limitations of the study

Despite the insights provided by this study, various limitations must be acknowledged. First, the relatively small sample size, consisting exclusively of elite weightlifters and cyclists, may limit the generalizability of the findings to other sports disciplines or broader athlete populations, including amateur or recreational athletes. Additionally, the lack of detailed dietary data from participants restricts the ability to correlate specific dietary patterns, such as the intake of carbohydrates, proteins, fats, and fibers, with the observed microbial and metabolomic profiles. Comprehensive dietary assessments would be essential for understanding how these factors influence the gut microbiota and metabolic outcomes in athletes.

Another limitation is the absence of direct correlation analyses between the gut microbiota, metabolomic data, and specific measures of physical performance. While potential links were identified, the study did not explicitly validate the role of microbial and metabolic factors in athletic performance.

Additionally, given the relatively small sample size, conducting separate analyses for weightlifters and cyclists could have compromised statistical power, making it challenging to detect significant microbiome-metabolome associations within each group. However, we recognize the potential value of such an approach and acknowledge that future studies with larger sample sizes may benefit from analyzing these groups independently to explore sport-specific microbial and metabolic signatures in greater detail.

Methodological differences, such as the use of untargeted versus targeted metabolomic analysis^73^, the inclusion of different sports^2^, and the assessment of acute versus chronic effects^72^, as well as the inclusion of both professional and nonprofessional athletes, may influence the comparability of our results with previous findings. Despite these variations, our lipidomic analysis provides complementary evidence to the microbial and metabolic data, offering a more integrated perspective on how the gut microbiota and its metabolic byproducts contribute to the physiological adaptations observed in elite athletes. Future research should continue to explore these relationships to further elucidate the mechanisms underlying athletic performance and to confirm possible connections between the host and microbiota through lipid-like molecules.

Furthermore, while this study employed multivariate approaches tailored to microbiome data analysis, we recognize that additional bioinformatic tools could provide further insights into microbiome-metabolome interactions. Methods such as mixOmics^74^ offer powerful techniques for dimension reduction and feature selection, which may enhance the integration of multi-omics datasets. However, given the constraints of our dataset, implementing mixOmics was beyond the scope of this study. Future research incorporating this approach could further refine the understanding of microbial and metabolic adaptations in different athletic disciplines. Our study also focused on a single time point (before competition), which limits the ability to capture temporal adaptations in the gut microbiota and metabolome. Longitudinal analyses, including samples taken during different phases such as pre- and post-competition or preparation periods, could provide deeper insights into these dynamics^19,27,32,75^. Finally, some of the proposed connections between gut microbial activity and metabolite production, particularly those involving lipid-like molecules, remain hypothetical and require further investigation for validation. These limitations highlight the need for future research to explore these relationships^27^ more explicitly and to address the gaps identified in this study.

## Methods

### Participants

Two groups of Colombian athletes, elite weightlifting athletes, and elite cyclist athletes, were recruited for this study. Both groups included athletes of professional Colombian leagues of weightlifting (men = 8; women = 8), practicing competitive sports for 7.5 ± 2.5 years, and cycling (men = 13), practicing competitive sports for 5.0 ± 1.6 years at the national and international levels. Both elite athlete groups were measured one month prior to an international competition. The inclusion/exclusion criteria applied in this study are detailed in our previous publication^19^. In brief, professional-level athletes engaged in competitive training according to their league’s schedule and affiliated with a Colombian sports league were invited to participate in this study. The specific requirements included a) adults (men and women) aged 18–45 years; b) athletes who had been actively training for at least the past six months; c) affiliation with a recognized sport league or federation; d) currently in the precompetitive stage; and e) being born in Colombia.

The exclusion criteria included the following: a) use of antibiotics, deworming agents, purgatives, or laxatives in the past six months; b) diagnosis of infectious diseases; c) presence of noncommunicable chronic diseases; d) diagnosis of metabolic diseases; e) medical history of cardiovascular disease, malignant neoplasm, chronic inflammatory disease, or psychiatric disorders; and f) presence of dyslipidemia, anemia, kidney disease, or smoking. Figure 1 presents a comprehensive overview of the methodological workflow used to study the gut microbiota and host metabolism in athletes.

All participants attended the laboratory appointment under similar conditions: 8 h of fasting and not having trained 24 h prior to attending the test center. We extracted blood from all the participants, collected the plasma, and stored it at −80 °C until analysis. Each participant provided fecal samples, which were stored at −80 °C for further analysis. The collection and storage procedures for the fecal samples followed established protocols^76^to ensure sample integrity and minimize contamination. The researchers labeled all samples with unique identifiers to maintain anonymity and track individual data throughout the study period^23^.

### Gut microbiome analysis through metagenomic analysis

#### DNA extraction and quality control

DNA extraction for compositional and functional analysis from the microbiome dataset was developed to ensure representation across a diverse range of organisms, and we utilized 29 stored stool samples. These samples included 16 from weightlifters and 13 from cyclists. The DNA extraction process employed the QIAamp PowerFecal DNA Kit by Qiagen, which is specifically designed for purifying DNA from samples rich in inhibitors (such as feces and intestinal contents). To determine purity and ensure precise quantification, a Nanodrop Kit (Implen, CA, USA) and a Qubit® 2.0 fluorometer (Life Technologies, CA, USA) were used.

#### Library preparation and sequencing

The protocol outlined for the Illumina Nextera XT DNA Library Preparation Kit was meticulously followed. Initially, samples were normalized to a concentration of 0.2 ng/μL per individual. This was followed by fragmentation and amplification steps. An index code was incorporated into the primer to differentiate various samples in the sequence data. After the quality of the library was verified, all the samples were subjected to paired-end sequencing via the Illumina HiSeq 2500 platform by a certified company. The sequencing depth was maintained at 2 GB for each sample, ensuring comprehensive coverage of genetic material. This depth allowed for robust analysis and identification of functional features such as gene family abundances.

#### Quality control and data processing for metagenomic analysis

The raw sequencing reads obtained from the Illumina platform were subjected to a series of quality control steps. First, quality assessment was performed via FastQC. Next, quality and adapter trimming were carried out via Trimmomatic to increase the reliability of the downstream analyses. To remove host-related reads, the trimmed data were aligned to the *H. sapiens*genome via Bowtie2. The resulting reads were then utilized to perform taxonomical and functional analyses via the BioBakery workflow^35^, which includes a suite of tools designed for comprehensive microbial community profiling and functional annotation^35^.

#### Taxonomic profiling via the MetaPhlAn4 and StrainPhlAn4 methods

Clean reads obtained from metagenomic samples, consisting of 16 samples from weightlifters and 13 samples from cyclists, were subjected to taxonomic profiling via MetaPhlAn4^77^ and StrainPhlAn4^78^. MetaPhlAn 4 utilizes a database of clade-specific marker genes derived from known microbial genomes. This approach allows for the identification and quantification of microbial taxa present in the samples on the basis of the alignment of cleaned reads against these markers. The relative abundance of each taxon in the metagenomic samples was determined from the alignment results generated by MetaPhlAn4^36^.

StrainPhlAn4 extends this analysis by performing strain-level profiling of microbial communities. It utilizes single-nucleotide variants (SNVs) in core genes to differentiate strains within taxonomic groups identified by MetaPhlAn4^37^. This provides a more detailed characterization of microbial populations, elucidating strain-level differences between samples. The taxonomic and strain-level profiles obtained from MetaPhlAn 4 and StrainPhlAn 4 were further analyzed statistically to assess differences in microbial composition and strain diversity between weightlifters and cyclists. The statistical significance of differences in microbial composition and strain diversity was evaluated via nonparametric tests due to the distribution of the data. MaAsLin2 was employed to perform multivariate analysis through a Negative Binomial regression model (NEGBIN)^20^.

To ensure the reliability of the taxonomic and strain-level assignments, the outputs were validated against established databases of microbial genomes (NCBI). Quality control measures were implemented throughout the analysis pipeline to validate the accuracy of taxonomic and strain-level profiling and to minimize potential biases. Taxonomic and strain-level profiles were visualized via Microbiome Analyst 2.0^79^ and R-based tools to illustrate the microbial community structure and strain diversity across samples.

### Functional profiling via the HumanN4 method

Following taxonomic profiling, functional annotation of the 29 metagenomic samples (16 from weightlifters and 13 from cyclists) was performed using **HUMAnN4**(The HMP Unified Metabolic Analysis Network, version 4)^35^. HUMAnN4 maps cleaned reads against a comprehensive database of reference sequences, including microbial genomes and gene families, to identify and quantify functional pathways and gene functions within the microbial community. The pipeline employs a tiered alignment strategy:

1. Rapid alignment of reads to a database of pangenomes representing microbial species.
2. Translation of unaligned reads into protein sequences, which are then searched against a database of protein families (UniRef90).

This approach allows for the quantification of microbial metabolic pathways and gene families, providing insights into the functional potential of the gut microbiota. The relative abundance of each pathway and gene family was normalized to account for variations in sequencing depth across samples. The MetaCyc database^42^ was used to annotate and interpret the metabolic pathways identified by HUMAnN4, offering additional insights into microbial metabolis.

To complement the HUMAnN4 analysis, or MaAsLin2^20^ was employed for differential abundance analysis of functional and taxonomic profiles. This pipeline is designed to identify statistically significant associations between microbial features (e.g., taxa, pathways, or genes) and metadata variables, such as athlete class (weightlifters vs. cyclists). The analysis was performed with the following configurations^20^:

Cumulative Sum Scaling (CSS) was applied to normalize the data, preventing biases due to variations in sequencing depth. No transformation (NONE) was applied to the normalized data, as the Negative Binomial regression model (NEGBIN) inherently accounts for variance stabilization. NEGBIN was used to assess the relationship between microbial features and the fixed effects of interest, including“Class”(weightlifters vs. cyclists) and"Weightlifting,"to evaluate differences in microbial composition and functional pathways between the two athlete groups. Random effects were not included, as the study design did not necessitate accounting for hierarchical or nested structures. To ensure the reliability of the identified associations, the Benjamini-Hochberg (BH) method was applied to control the false discovery rate (FDR) and adjust p-values for multiple comparisons. This approach allowed us to identify microbial taxa, pathways, and gene families that were significantly differentially abundant between weightlifters and cyclists, providing insights into the functional and taxonomic differences in the gut microbiota of these athlete groups.

### Untargeted metabolomic and lipidomic analyses of fecal and plasma samples

In this study, we employed untargeted metabolomic and lipidomic analyses to investigate the metabolic profiles of fecal and plasma samples from weightlifters (16 samples) and cyclists (13 samples). The lipidomic profiles of the plasma samples were specifically analyzed to characterize the lipid profiles. Various analytical platforms, including liquid chromatography‒mass spectrometry (LC‒MS) and gas chromatography‒mass spectrometry (GC‒MS), have been utilized to analyze the metabolites present in these samples comprehensively. This approach allows exploration of metabolic pathways and lipid compositions, providing insights into metabolic differences between athletes from different disciplines.

### Metabolomic analysis of fecal samples via LC‒MS and GC‒MS

Sample preparation for fecal metabolomic analysis via reversed-phase liquid chromatography‒quadrupole time‒of‒flight mass spectrometry (RP‒LC‒MS‒QTOF) began with lyophilization to reduce the moisture content, followed by precise weighing. Each sample was allocated for subsequent processing, extraction, and identification via an optimized protocol tailored for untargeted metabolomic analysis^80^.

Fecal samples (50 mg) were weighed and mixed with 1000 µL of MeOH, vortexed for 15 min, and then sonicated for 15 min. After another vortex step (5 min), the samples were centrifuged at 16,000 rpm and 4 °C for 10 min. Subsequently, 100 µL of the extract was collected for further analysis via LC-QTOF-MS.

The samples were analyzed via an Agilent Technologies 1260 liquid chromatography system coupled to a quadrupole time-of-flight Q-TOF 6545 mass analyzer with electrospray ionization. A 1 µL aliquot of each sample was injected onto a C_18_ column (InfinityLab Poroshell 120 EC-C_18_ 100 × 2.1 mm, 1.9 µm) at 40 °C via gradient elution consisting of 0.1% (v/v) formic acid in Milli-Q water (Phase A) and 0.1% (v/v) formic acid in acetonitrile (Phase B) at a constant flow rate of 0.4 mL/min. Mass spectrometry detection was conducted in positive electrospray ionization (ESI) mode, scanning from 50 to 1100 m*/z* in both full scan and MS/MS modes. Throughout the analysis, mass correction was performed using two reference masses: *m/z* 121.0509 (C_5_H_4_N_4_) and *m/z* 922.0098 (C_18_H_18_O_6_N_3_P_3_F_24_).

From the extracts prepared for RP-LC/MS-QTOF analysis, 20 µL was dried in a SpeedVac for 1 h. Then, 10 µL of *O-*methoxime in pyridine (15 mg/mL) was added, and the mixture was vortexed for 5 min and incubated in the dark at room temperature for 16 h. Silylation was performed by adding 10 µL of *N,O-*bis(trimethylsilyl)trifluoroacetamide (BSTFA) with 1% trimethylchlorosilane (TMCS), followed by vortexing for 5 min and incubation at 70 °C for 1 h. After cooling to room temperature, 200 µL of methyl stearate (5 mg/L) was added as an internal standard. Data acquisition was performed on an Agilent Technologies 7890B GC coupled with an Agilent Technologies 7250 GC/Q-TOF mass spectrometer. A 1 µL aliquot of derivatized sample was injected at a split ratio of 30:1 onto an HP-5MS column (30 m, 0.25 mm, 0.25 µm) (Agilent Technologies) at a constant flow rate of 0.7 mL/min. The oven temperature was programmed from 60 °C (1 min) at 10 °C/min to 325 °C (10 min). Mass spectra were recorded at 70 eV in full-scan mode with values ranging from 50 to 600 m*/z*. The transfer line, ion source, and quadrupole temperatures were maintained at 280 °C, 230 °C, and 150 °C, respectively.

### Metabolomic analysis of the plasma samples via GC-QTOF-MS and LC-QTOF-MS.

Two hundred microliters of plasma was mixed with 600 µL of cold MeOH, vortexed, and stored at −20 °C for 20 min before centrifugation. For GC-QTOF-MS analysis, 50 µL of the plasma extract preparation was dried and subjected to derivatization following a previously described procedure.

LC-QTOF-MS analysis of the plasma samples was conducted via an Agilent Technologies 1260 liquid chromatography system coupled to a 6545 Q-TOF time‒of‒flight quadrupole mass analyzer with electrospray ionization, following the same methodology as previously described for the fecal samples.

### Lipidomic analysis of the plasma samples via LC‒QTOF‒MS

The process involved extracting 100 µL of plasma and then combining it with 350 µL of cold MeOH (−20 °C) and an additional 350 µL of MTBE. Next, they were subjected to vortexing for 5 min and subsequently centrifuged at 13,000 rpm and 20 °C for a period of 10 min. For further analysis, a total of 100 µL of the supernatant was transferred into an Eppendorf tube.

Lipidomic analysis of the plasma samples was performed via an Agilent Technologies 1260 Liquid Chromatography system, which was coupled to a 6545 Q-TOF time-of-flight quadrupole mass analyzer with electrospray ionization. A volume of 1 µL of the extracts was introduced into a C_18_ column (InfinityLab Poroshell 120 100 × 3.0 mm, 2.7 µm) maintained at 50 °C. The elution process followed a gradient consisting of 10 mM ammonium acetate at a ratio of 90:10 (ACN:H_2_O) (Phase A) and 10 mM ammonium acetate at a ratio of 20:30:50 (ACN:MeOH:IPA) (Phase B) with a constant flow rate of 0.4 mL/min. Mass spectrometry detection was conducted in positive electrospray ionization (ESI) mode in both full-scan mode and MS/MS mode, covering the mass range of 40–2000 m*/z*. In the course of the analysis, the reference masses utilized for mass correction were *m/z* 121.0509 (C_5_H_4_N_4_) and *m/z* 922.0098 (C_18_H_18_O_6_N_3_P_3_F_24_).

### Quality control of metabolomic and lipidomic analyses

Quality assurance and control practices in metabolomic analyses by MS followed the recommendations of the international mQACC consortium^81^. These practices included the analysis of quality control (QC) samples, reference standards, and blanks. The QC samples were prepared by pooling equal volumes of the samples to be analyzed and were extracted and analyzed in the same manner as the study samples. To assess the reproducibility and stability of the analytical platforms used, QC samples were analyzed before the analysis sequence to balance the chromatographic system and were injected every five samples throughout the sequence.

### Data processing and statistical analysis of metabolomic and lipidomic data

The raw data from the LC‒QTOF‒MS system were processed via Agilent MassHunter Profinder B.10.0 software for deconvolution, alignment, and integration. For the GC‒QTOF‒MS data, these steps were carried out with Agilent Unknowns Analysis B.10.0, MassProfiler Professional B.15.0, and Agilent Mass Hunter Quantitative Analysis B.10.0. The data from all the platforms were subsequently thoroughly analyzed. A presence and reproducibility filter was applied, retaining only metabolites found in at least 80% of the samples within the same group and exhibiting a coefficient of variation (CV, %) below 20% in the QC samples for the LC data (30% for the GC data). These filtered metabolites were then selected for statistical analysis.

To identify molecular features with statistically significant differences between weightlifters and cyclists, both univariate (UVA) and multivariate (MVA) statistical analyses were performed via the MetaboAnalyst server. For the UVA analysis, p values were computed via nonparametric tests. In MVA, principal component analysis (PCA) was initially employed as an unsupervised method to evaluate data quality and sample distribution. This was followed by the application of supervised orthogonal partial least squares discriminant analysis (OPLS-DA) models to identify the molecular features driving group separation. The effectiveness and precision of the OPLS-DA models were assessed via R^2^, Q^2^, permutation tests, and cross-validation analysis of variance. Statistically significant features were selected on the basis of the following criteria: (1) UVA—p value < 0.05 and (2) MVA—variance important in projection (VIP) > 1.

### Metabolite identification

To annotate significant features from liquid chromatography, a variety of parameters have been employed. These included confirming retention times and the potential for adduct formation, comparing high-resolution mass data with database entries via the CEU Mass Mediator tool (http://ceumass.eps.uspceu.es, accessed in 2023), and deriving theoretical formulas on the basis of isotopic distributions. MS/MS data were cross-referenced with spectra in MS-DIAL 4.80 (http://prime.psc.riken.jp/compms/msdial/main.html) and Lipid Annotator software v10.0. Manual interpretation of the MS/MS spectra was also conducted. For GC analysis, compound identification was achieved by comparing mass spectra and FAME retention indices with entries in the Fiehn GC‒MS Metabolomics RTL (Retention Time Locked) Library 2013 (https://pubs.acs.org/doi/abs/10.1021/ac9019522). Finally, identification levels were assigned for each platform according to the guidelines provided by the Metabolomics Standards Initiative as described by Blaženović et al. (2018). The lowest level of annotation was achieved with a match to the exact mass (4), followed by confirmation of the molecular formula (3), identification of specific compound fragment signals (2), and, ultimately, confirmation at the standard level (1)^44^.

### Integrative analysis of gut microbiome functional profiling

In this study, state-of-the-art bioinformatic tools were employed to investigate the functional potential of the gut microbiome in weightlifters and cyclists^35,45,46,48^. An integrative analysis of the gut microbiome and hypothetical metabolites, reviewed from the literature, produced by the gut microbiota was used to create a multiomics integration under a knowledge-driven approach, following the methodology proposed by Ewald and collaborators (web-based multiomics integration using the Analyst software suite)^46^. The Analyst software suite, specifically OmicsNet 2.0, was utilized for this purpose^45^.

The model construction followed the approach described by Ewald et al., wherein each omics data type was analyzed separately. After statistical analysis, significant species identified by MetaPhlAn4 and metabolites identified from fecal and plasma metabolomic analysis were selected to create an integrative network. This multiomics integration provided a comprehensive view of the interactions between the gut microbiome and metabolomic profiles, enabling a deeper understanding of the functional dynamics in weightlifters and cyclists.

## Supplementary Information

Supplementary Information 1.

Supplementary Information 2.

Supplementary Information 3.

## Data Availability

The datasets generated and analyzed during the current study are available in the European Nucleotide Archive (ENA) under the Project PRJEB82418 (ERP166105) (https://www.ebi.ac.uk/ena/browser/view/PRJEB82418). Metabolomics data is available at the NIH Common Fund’s National Metabolomics Data Repository (NMDR) website, the Metabolomics Workbench, https://www.metabolomicsworkbench.org, where it has been assigned Project ID ST003710.
