# Supplementary material for: Integrating metagenomics and metabolomics to study the gut microbiome and host relationships in sports across different energy systems
Source: Sci Rep. 2025 May 2;15:15356. doi: 10.1038/s41598-025-98973-2 (PMC12048592; doi:10.1038/s41598-025-98973-2)
Supplement: Supplementary file 1 — Supplementary Information 1. [file 41598_2025_98973_MOESM1_ESM.pdf]

Phylogenetic Trees of *Prevotella copri*, *Bacteroides finegoldii*, and *Bacteroides caccae* in Athletes.

A) *Prevotella copri*

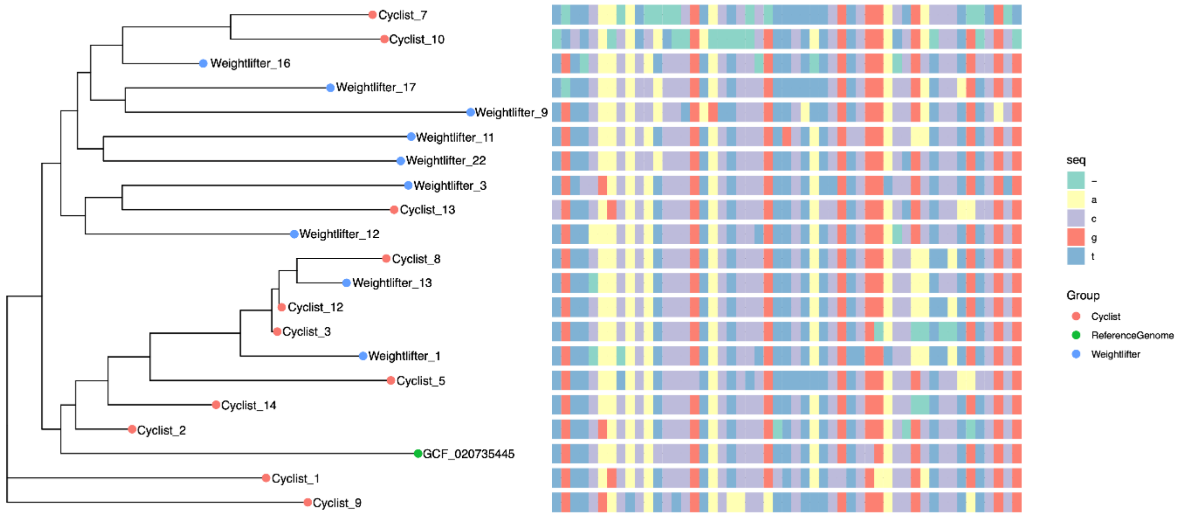

C) *Bacteroides finegoldii*

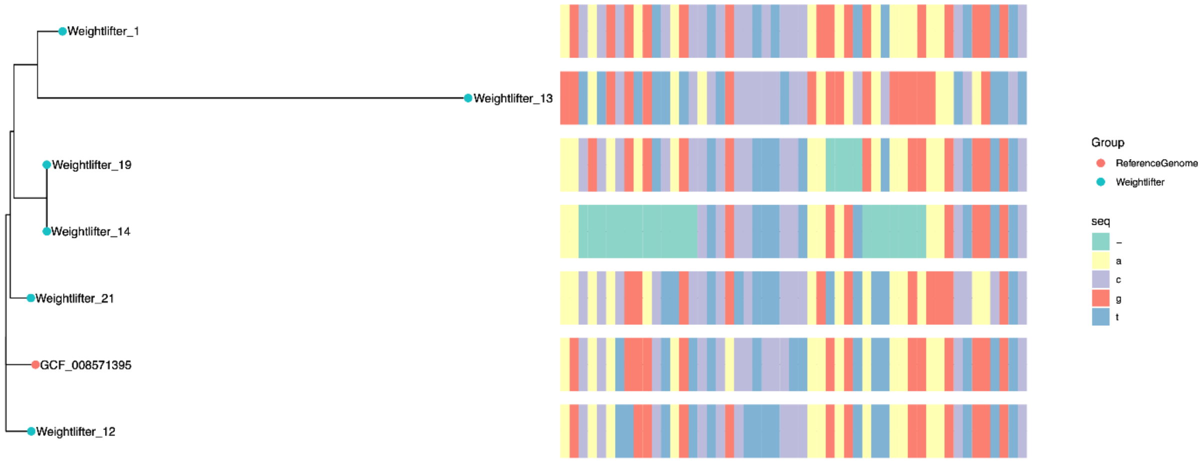

C) ***Bacteroides caccae***

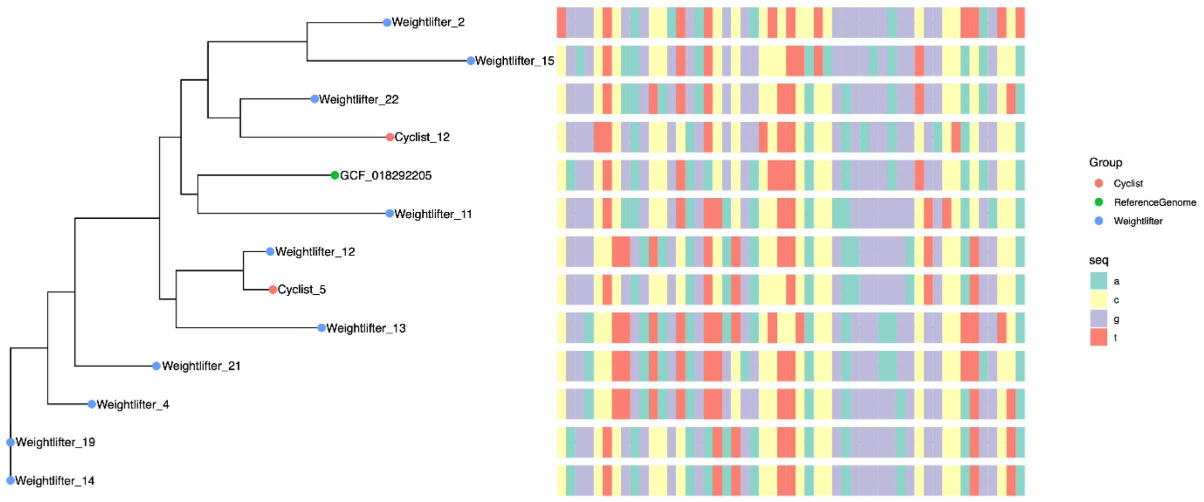

Figure S1: Phylogenetic Trees of Specific Strains Constructed with StrainPhlAn.

The phylogenetic trees were constructed using StrainPhlAn for A) *Prevotella copri*, B) *Bacteroides finegoldii*, and C) *Bacteroides caccae*. Sequences from the athlete samples were aligned to reference genomes for comparative purposes. The cladistic distance is represented on the X-axis. The reference genome strains are included in each tree to allow for comparative phylogenetic analysis, highlighting differences in strain-level variation between the studied groups (weightlifters and cyclists). These analyses provide insights into the potential functional impacts of specific bacterial strains on host performance and metabolism.

Quality Control of GC-MS and LC-MS Data in Plasma and Fecal Samples

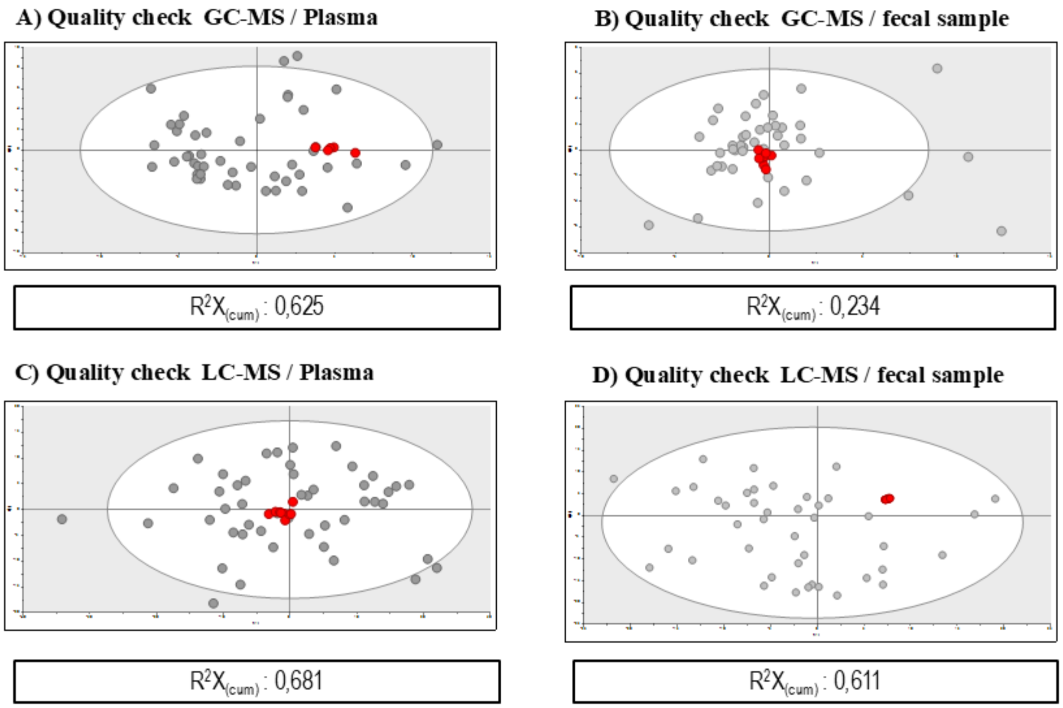

Figure S2: Quality Check of GC-MS and LC-MS Analyses in Plasma and Fecal Samples.

The clustering of Quality Control (QC) samples is shown for both plasma and fecal samples using GC-MS and LC-MS techniques. A) GC-MS analysis of plasma samples shows a cumulative R^2X of 0.625, indicating good clustering of the QC samples (in red). B) GC-MS analysis of fecal samples with a cumulative R^2X of 0.234, showing more dispersed clustering. C) LC-MS analysis of plasma samples, with a cumulative R^2X of 0.681, showing tighter clustering of QC samples. D) LC-MS analysis of fecal samples, with a cumulative R^2X of 0.611, also demonstrating a strong clustering of QC samples. The results indicate consistent analytical performance across the methods used, especially for plasma samples.

Metabolomic Heatmap Showing Key Differences in Plasma Metabolites Between Weightlifting and Cycling Athletes.

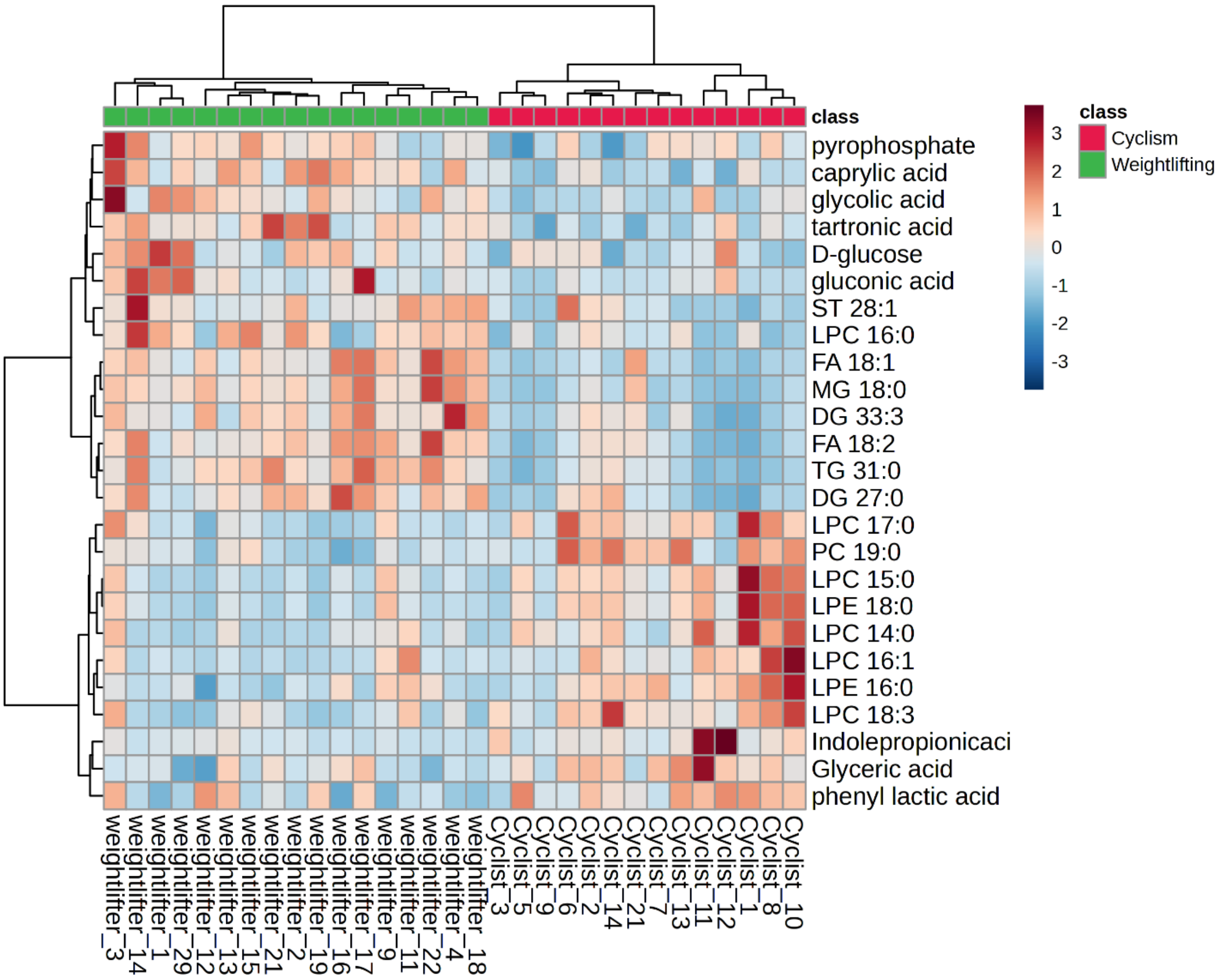

Figure S3: Hierarchical Clustering Heatmap of Plasma Metabolomic Signatures in Weightlifters and Cyclists.

The heatmap illustrates the hierarchical clustering of plasma metabolites from weightlifting athletes (WA) and cycling athletes (CA). The color scale indicates the relative abundance of metabolites, with blue representing lower abundance and red representing higher abundance. Key metabolic signatures, such as pyrophosphate, caprylic acid, LPC 16:0, and others, were identified as discriminative between the WA and CA groups. The clustering pattern highlights group-specific metabolic pathways potentially related to the distinct energy demands of each sport. Each metabolite was selected based on its contribution to group separation using a feature selection approach focused on relevance to performance and metabolic adaptation.

OPLS-DA Lipidomic Analysis and LION Network Representation of Lipid Pathways in Cyclists vs Weightlifters.

4A) OPLS-DA lipidomics

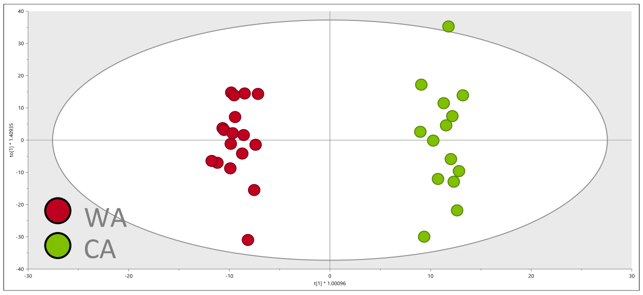

R2X(cum): 0.626 R2Y(cum): 0.984 Q2(cum): 0.808
p: 3.14953e-05

4B) Correlation pattern of TG

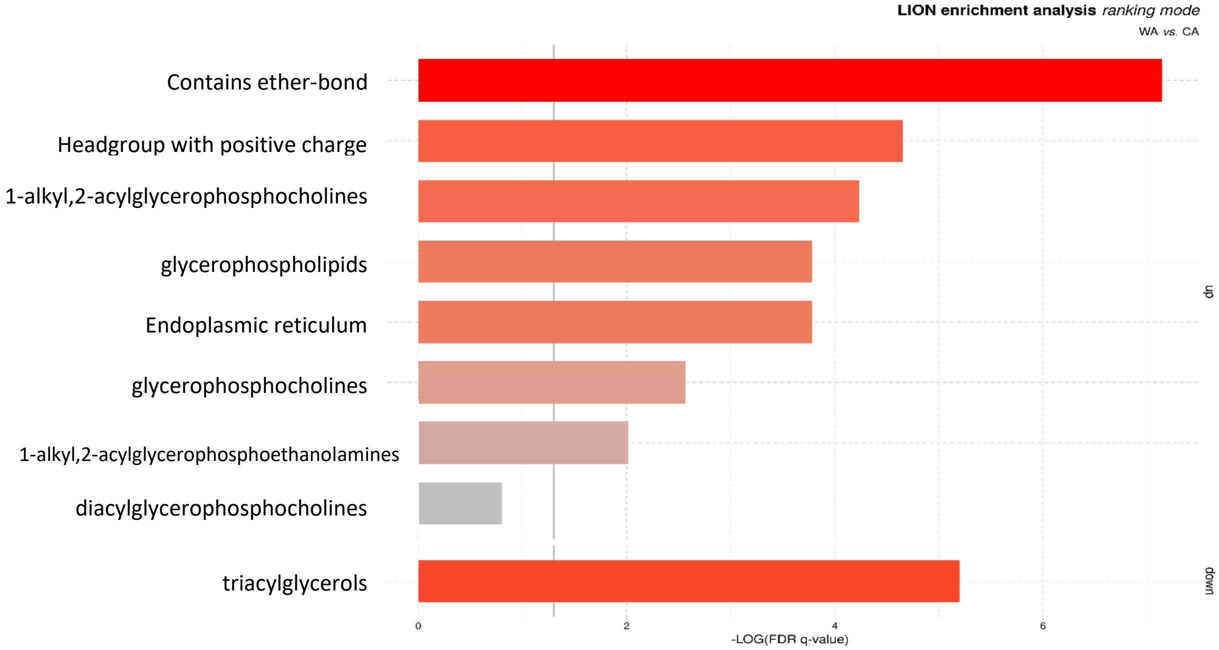

4C) LION network

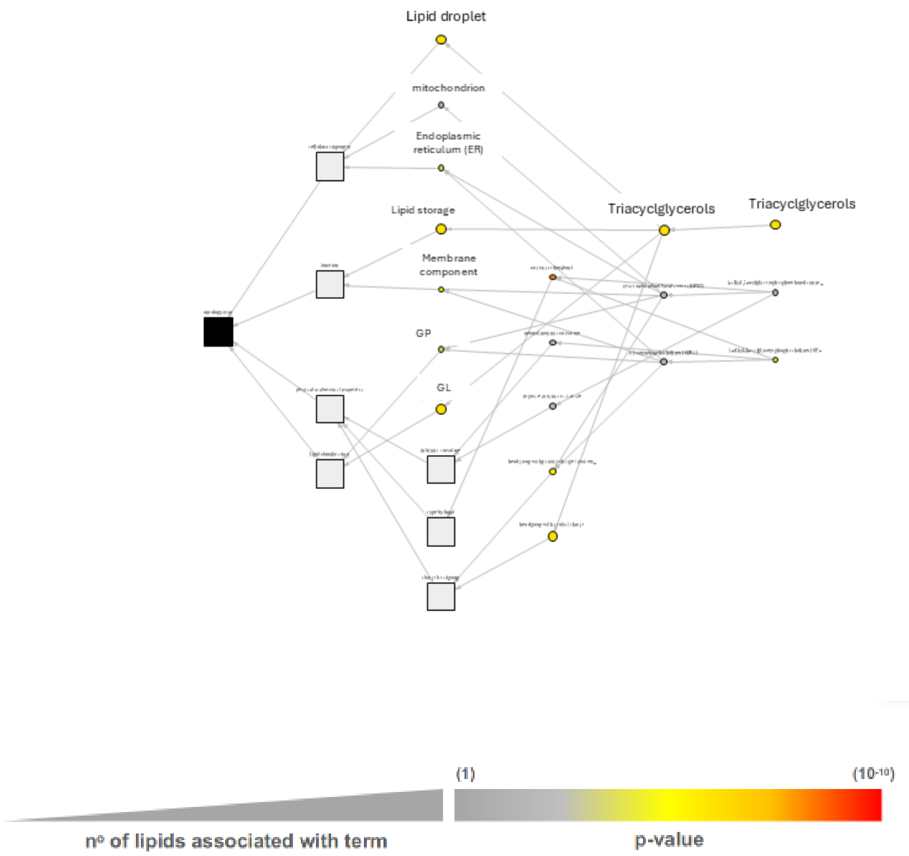

Figure S4: Lipidomic Profiling and Enrichment Analysis of Plasma Samples from Weightlifting and Cycling Athletes.

(4A) The Orthogonal Partial Least Squares Discriminant Analysis (OPLS-DA) demonstrates a robust separation between the lipidomic profiles of weightlifting athletes (WA) and cycling athletes (CA). This model's performance metrics show excellent fit and predictivity with R2X(cum): 0.626, R2Y(cum): 0.984, and Q2(cum): 0.808, along with a significant p-value (3.14953e-05), indicating substantial differences in lipid profiles between the two groups.

(4B) Correlation pattern of triglycerides (TG) highlights significant differences between groups, where TG species were markedly elevated in cyclists, reflecting increased reliance on lipid metabolism during endurance exercise. Lipid enrichment analysis via LION/web further identified pathways associated with lipid droplet formation, storage, and glycolipid synthesis.

(4C) LION network analysis showing the upregulated pathways related to lipid metabolism and energy storage, particularly in the cycling group, further supporting the metabolic adaptations linked to endurance training. Yellow nodes indicate significant lipid-associated terms, with

colors corresponding to the p-value, and the size of the nodes representing the number of lipids associated with each term.

Additional functional pathways associated with gut microbiota in professional road cyclists and weightlifters

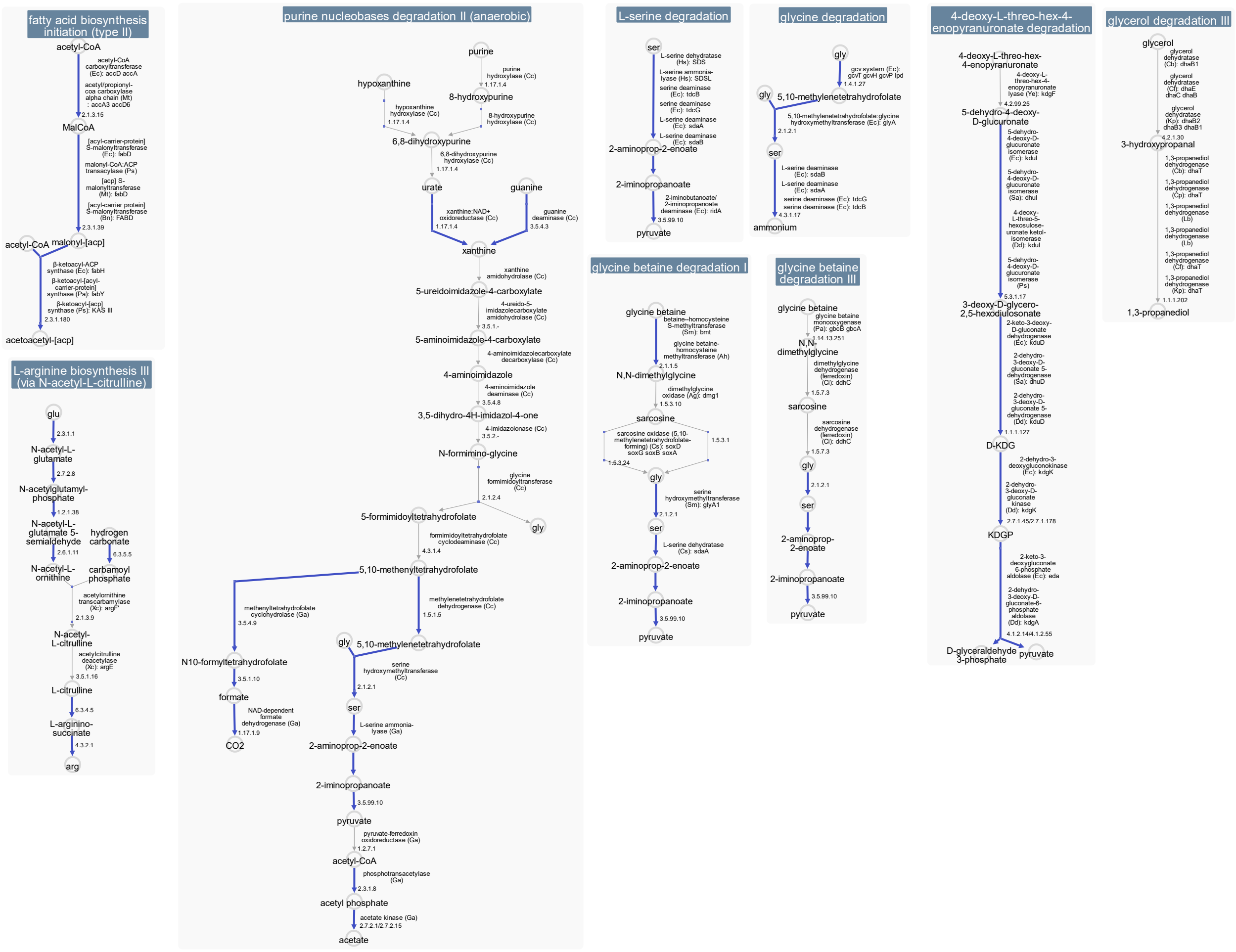

Figure S5: This pathway collage expands on the pathways presented in Figure 3B by providing additional superpathways selected for their biological relevance to the study's objectives and potential implications in athletic performance. These include pathways such as Amino Acid Biosynthesis, Generation of Precursor Metabolites and Energy, Alcohol Degradation, Carbohydrate Degradation, Fatty Acid and Lipid Biosynthesis, Secondary Metabolite Biosynthesis, Nucleoside and Nucleotide Degradation, Amide, Amidine, Amine, and Polyamine Degradation, as well as Amino Acid and Carbohydrate Degradation. Metabolites are represented in circles, and enzyme and gene labels are provided, including EC codes. Blue edges represent reactions identified through metagenomic data. These supplementary pathways offer a broader context, complementing the main figure and providing further insights into the metabolic potential observed in the athlete groups. Figure created with MetaCyc.
